# Supplementary material for: A widely employed germ cell marker is an ancient disordered protein with reproductive functions in diverse eukaryotes
Source: eLife. 2016 Oct 8;5:e19993. doi: 10.7554/eLife.19993 (PMC5098910; doi:10.7554/eLife.19993)
Supplement: Figure 4—figure supplement 1—source data 1. — DOI: http://dx.doi.org/10.7554/eLife.19993.016 [file elife-19993-fig4-figsupp1-data1.docx]

GCNA PROTEASE HMM

HMMER3/f [3.1b1 | May 2013]

NAME GCNAsprtProteaseonlyUPDATE

LENG 99

ALPH amino

RF no

MM no

CONS yes

CS no

MAP yes

DATE Mon Mar 9 15:10:01 2015

NSEQ 14

EFFN 0.967285

CKSUM 1497135770

STATS LOCAL MSV -9.5317 0.71785

STATS LOCAL VITERBI -10.3880 0.71785

STATS LOCAL FORWARD -4.1105 0.71785

HMM A C D E F G H I K L M N P Q R S T V W Y

m->m m->i m->d i->m i->i d->m d->d

COMPO 2.47439 3.98252 3.05804 2.65636 3.38600 3.02880 3.31665 2.88803 2.60770 2.45708 3.63452 3.07362 3.67603 3.09023 2.91773 2.65917 2.80558 2.68837 3.76145 3.60616

2.68579 4.42229 2.77524 2.73127 3.46358 2.40517 3.72499 3.29358 2.67745 2.69359 4.24694 2.90351 2.73744 3.18150 2.89805 2.37891 2.77513 2.98510 4.58481 3.61507

0.42253 1.34352 2.48067 0.20598 1.68118 0.00000 *

1 2.48556 4.87281 2.92232 2.25793 4.14059 3.40736 3.61244 3.16662 2.19917 3.14135 3.94763 2.92565 3.81006 2.30483 2.80340 2.27637 2.85232 3.22201 5.35828 4.01766 2 k - - -

2.68618 4.42225 2.77519 2.73123 3.46354 2.40513 3.72494 3.29354 2.67741 2.69355 4.24690 2.90347 2.73739 3.18146 2.89801 2.37887 2.77519 2.98518 4.58477 3.61503

0.02599 4.05892 4.78127 0.61958 0.77255 0.52247 0.89907

2 2.47995 3.81276 2.70397 2.27811 4.23790 3.39292 3.31083 3.68198 2.14085 3.22837 4.00800 2.88876 3.78642 2.59227 2.49977 2.44178 2.83016 3.30989 5.41656 4.04853 3 k - - -

2.68618 4.42225 2.77519 2.73123 3.46354 2.40513 3.72494 3.29354 2.67741 2.69355 4.24690 2.90347 2.73739 3.18146 2.89801 2.37887 2.77519 2.98518 4.58477 3.61503

0.02526 4.08711 4.80945 0.61958 0.77255 0.45153 1.01240

3 3.04633 4.42136 4.61184 4.04977 2.24660 4.19213 4.27764 2.12833 3.90854 1.19365 3.04603 4.18521 4.47580 4.02687 4.01341 3.50750 3.26980 2.42432 4.64353 2.81465 4 l - - -

2.68618 4.42225 2.77519 2.73123 3.46354 2.40513 3.72494 3.29354 2.67741 2.69355 4.24690 2.90347 2.73739 3.18146 2.89801 2.37887 2.77519 2.98518 4.58477 3.61503

0.02399 4.13790 4.86025 0.61958 0.77255 0.48576 0.95510

4 2.48936 4.28238 3.84811 3.29479 2.22812 3.74314 3.84236 2.72241 3.19918 2.17363 3.35987 3.57647 4.11093 3.01720 3.45788 3.01954 2.95626 2.53966 4.48492 2.11069 5 y - - -

2.68618 4.42225 2.77519 2.73123 3.46354 2.40513 3.72494 3.29354 2.67741 2.69355 4.24690 2.90347 2.73739 3.18146 2.89801 2.37887 2.77519 2.98518 4.58477 3.61503

0.02399 4.13790 4.86025 0.61958 0.77255 0.48576 0.95510

5 2.22095 5.07656 2.49163 2.22514 4.39199 3.38867 3.60042 3.85447 2.02741 3.36569 4.13137 2.86790 3.80058 2.50062 2.69734 2.61839 2.74388 3.45233 5.52336 4.13370 6 k - - -

2.68618 4.42225 2.77519 2.73123 3.46354 2.40513 3.72494 3.29354 2.67741 2.69355 4.24690 2.90347 2.73739 3.18146 2.89801 2.37887 2.77519 2.98518 4.58477 3.61503

0.02399 4.13790 4.86025 0.61958 0.77255 0.48576 0.95510

6 2.60907 4.61047 3.15360 2.37007 3.80252 3.12373 3.69354 2.91653 2.52779 2.32429 3.37911 3.07581 3.86988 2.68660 2.50662 2.70554 2.83871 2.91451 5.14380 3.85715 7 l - - -

2.68618 4.42225 2.77519 2.73123 3.46354 2.40513 3.72494 3.29354 2.67741 2.69355 4.24690 2.90347 2.73739 3.18146 2.89801 2.37887 2.77519 2.98518 4.58477 3.61503

0.02399 4.13790 4.86025 0.61958 0.77255 0.48576 0.95510

7 3.28222 4.59300 4.65355 4.21380 1.76807 4.24102 3.66315 3.01454 4.04903 1.66013 3.62286 4.07301 4.55735 4.07236 4.08957 3.57000 3.50380 2.90356 2.99831 1.56187 8 y - - -

2.68618 4.42225 2.77519 2.73123 3.46354 2.40513 3.72494 3.29354 2.67741 2.69355 4.24690 2.90347 2.73739 3.18146 2.89801 2.37887 2.77519 2.98518 4.58477 3.61503

0.02399 4.13790 4.86025 0.61958 0.77255 0.48576 0.95510

8 2.77555 5.16865 2.32184 2.07893 4.46517 3.34681 3.71359 3.91849 2.57219 3.47142 4.28052 1.64175 3.85400 2.85795 3.08001 2.73576 3.03250 3.04389 5.66127 4.25089 9 n - - -

2.68618 4.42225 2.77519 2.73123 3.46354 2.40513 3.72494 3.29354 2.67741 2.69355 4.24690 2.90347 2.73739 3.18146 2.89801 2.37887 2.77519 2.98518 4.58477 3.61503

0.02399 4.13790 4.86025 0.61958 0.77255 0.48576 0.95510

9 2.39419 5.02123 2.48137 2.21392 4.32665 3.39168 3.22332 3.78409 2.26556 3.30840 4.07499 2.87667 3.79024 2.70294 2.43199 2.50513 2.74120 3.17723 5.47753 4.09350 10 e - - -

2.68618 4.42225 2.77519 2.73123 3.46354 2.40513 3.72494 3.29354 2.67741 2.69355 4.24690 2.90347 2.73739 3.18146 2.89801 2.37887 2.77519 2.98518 4.58477 3.61503

0.02399 4.13790 4.86025 0.61958 0.77255 0.48576 0.95510

10 2.66647 5.01894 2.93092 2.22758 4.32985 3.42993 3.60724 3.76905 2.17026 3.29763 4.08149 2.92357 3.82796 2.47522 2.37079 2.24229 2.45901 3.39372 5.46495 4.11069 11 k - - -

2.68618 4.42225 2.77519 2.73123 3.46354 2.40513 3.72494 3.29354 2.67741 2.69355 4.24690 2.90347 2.73739 3.18146 2.89801 2.37887 2.77519 2.98518 4.58477 3.61503

0.02399 4.13790 4.86025 0.61958 0.77255 0.48576 0.95510

11 3.12769 4.47024 4.81348 4.34447 3.66384 4.41941 5.07546 1.66186 4.21682 2.18861 3.48728 4.54603 4.78323 4.50933 4.41986 3.82702 3.41208 0.86813 5.62175 4.38942 12 v - - -

2.68618 4.42225 2.77519 2.73123 3.46354 2.40513 3.72494 3.29354 2.67741 2.69355 4.24690 2.90347 2.73739 3.18146 2.89801 2.37887 2.77519 2.98518 4.58477 3.61503

0.02399 4.13790 4.86025 0.61958 0.77255 0.48576 0.95510

12 3.01908 3.41299 4.50015 4.06542 1.04172 3.93469 4.08132 2.69354 3.91426 2.27272 3.50252 4.07546 4.40567 4.07436 4.03214 3.35677 3.32964 2.59790 4.40652 2.83533 13 f - - -

2.68618 4.42225 2.77519 2.73123 3.46354 2.40513 3.72494 3.29354 2.67741 2.69355 4.24690 2.90347 2.73739 3.18146 2.89801 2.37887 2.77519 2.98518 4.58477 3.61503

0.02399 4.13790 4.86025 0.61958 0.77255 0.48576 0.95510

13 2.79315 5.30689 1.84685 2.12985 4.61761 2.61030 3.69621 4.10887 2.55631 3.60327 4.38876 2.59838 3.84222 2.29505 3.08789 2.47628 3.04750 3.68539 5.75839 4.31384 14 d - - -

2.68618 4.42225 2.77519 2.73123 3.46354 2.40513 3.72494 3.29354 2.67741 2.69355 4.24690 2.90347 2.73739 3.18146 2.89801 2.37887 2.77519 2.98518 4.58477 3.61503

0.02399 4.13790 4.86025 0.61958 0.77255 0.48576 0.95510

14 2.47573 5.07339 2.61747 2.31703 4.38628 2.54212 3.61458 3.84844 2.27672 3.36741 4.13805 2.41730 3.80350 2.50952 2.71749 2.53030 2.88968 3.44983 5.53323 4.14087 15 k - - -

2.68618 4.42225 2.77519 2.73123 3.46354 2.40513 3.72494 3.29354 2.67741 2.69355 4.24690 2.90347 2.73739 3.18146 2.89801 2.37887 2.77519 2.98518 4.58477 3.61503

0.02399 4.13790 4.86025 0.61958 0.77255 0.48576 0.95510

15 2.51827 4.89603 3.03342 2.35480 4.16730 3.45788 3.62173 3.19317 1.92762 3.15514 3.43402 2.97402 3.84310 2.53587 2.34950 2.67214 2.87856 3.24670 5.36262 4.03624 16 k - - -

2.68618 4.42225 2.77519 2.73123 3.46354 2.40513 3.72494 3.29354 2.67741 2.69355 4.24690 2.90347 2.73739 3.18146 2.89801 2.37887 2.77519 2.98518 4.58477 3.61503

0.02399 4.13790 4.86025 0.61958 0.77255 0.48576 0.95510

16 2.95517 4.45785 4.19385 3.66719 2.69955 4.00515 4.27367 2.46933 3.54800 1.07277 3.12473 3.94091 3.37027 3.78734 3.76574 3.32864 3.20137 2.46369 4.86557 3.57936 17 l - - -

2.68618 4.42225 2.77519 2.73123 3.46354 2.40513 3.72494 3.29354 2.67741 2.69355 4.24690 2.90347 2.73739 3.18146 2.89801 2.37887 2.77519 2.98518 4.58477 3.61503

0.06115 4.13790 3.13819 0.61958 0.77255 0.48576 0.95510

17 2.08245 4.29548 3.37408 3.01539 4.25025 3.07213 4.11851 3.63175 3.04504 3.32100 4.15351 3.27383 1.58906 3.35983 3.39728 2.20576 2.34161 3.15777 5.58483 4.34830 18 p - - -

2.68618 4.42225 2.77519 2.73123 3.46354 2.40513 3.72494 3.29354 2.67741 2.69355 4.24690 2.90347 2.73739 3.18146 2.89801 2.37887 2.77519 2.98518 4.58477 3.61503

0.02489 4.10164 4.82398 0.61958 0.77255 0.52729 0.89209

18 2.24886 4.95238 2.83777 1.90202 4.24368 3.39156 3.62711 3.67776 2.24785 3.23939 4.03574 2.90173 3.81345 2.62219 2.85028 2.42350 2.88049 3.12842 5.44016 4.08010 19 e - - -

2.68627 4.42234 2.77515 2.73103 3.46363 2.40522 3.72504 3.29363 2.67707 2.69349 4.24639 2.90356 2.73749 3.18156 2.89810 2.37875 2.77529 2.98528 4.58486 3.61513

0.14976 2.03219 4.82398 1.28028 0.32567 0.46064 0.99663

19 2.51826 5.29179 1.83809 2.05060 4.60459 3.34209 3.68152 4.09190 2.01390 3.58309 4.36447 2.54581 3.83949 2.80840 3.02927 2.71607 3.03040 3.66906 5.73277 4.29656 25 d - - -

2.68618 4.42225 2.77519 2.73123 3.46354 2.40513 3.72494 3.29354 2.67741 2.69355 4.24690 2.90347 2.73739 3.18146 2.89801 2.37887 2.77519 2.98518 4.58477 3.61503

0.02399 4.13790 4.86025 0.61958 0.77255 0.48576 0.95510

20 3.22354 4.53920 4.95818 4.37785 2.78500 4.49209 4.80804 1.72492 4.24854 1.17955 2.27963 4.56274 4.69853 4.31356 4.32045 3.82888 3.44402 2.09213 5.12309 4.05402 26 l - - -

2.68618 4.42225 2.77519 2.73123 3.46354 2.40513 3.72494 3.29354 2.67741 2.69355 4.24690 2.90347 2.73739 3.18146 2.89801 2.37887 2.77519 2.98518 4.58477 3.61503

0.07660 4.13790 2.85112 0.61958 0.77255 0.48576 0.95510

21 2.61741 5.01376 2.69917 2.04207 4.31701 2.99235 3.59092 3.77263 2.22388 3.30193 4.07418 2.86283 2.92814 2.70803 2.68100 2.51009 2.85140 3.18004 5.47600 4.09228 27 e - - -

2.68618 4.42225 2.77519 2.73123 3.46354 2.40513 3.72494 3.29354 2.67741 2.69355 4.24690 2.90347 2.73739 3.18146 2.89801 2.37887 2.77519 2.98518 4.58477 3.61503

0.02527 4.08657 4.80892 0.61958 0.77255 0.45121 1.01297

22 3.22514 4.49993 5.01561 4.49187 3.51608 4.63647 5.12851 1.15099 4.38231 1.71370 3.30422 4.70402 4.87747 4.58429 4.54161 4.01252 3.47252 1.43968 5.53169 4.37430 28 i - - -

2.68618 4.42225 2.77519 2.73123 3.46354 2.40513 3.72494 3.29354 2.67741 2.69355 4.24690 2.90347 2.73739 3.18146 2.89801 2.37887 2.77519 2.98518 4.58477 3.61503

0.02399 4.13790 4.86025 0.61958 0.77255 0.48576 0.95510

23 2.59142 4.47156 3.26358 2.70439 3.65118 3.21996 3.74801 2.78071 2.53745 2.70875 3.57920 2.99099 3.89470 2.99671 2.79574 2.59080 2.47723 2.17528 5.04182 3.77882 29 v - - -

2.68618 4.42225 2.77519 2.73123 3.46354 2.40513 3.72494 3.29354 2.67741 2.69355 4.24690 2.90347 2.73739 3.18146 2.89801 2.37887 2.77519 2.98518 4.58477 3.61503

0.02399 4.13790 4.86025 0.61958 0.77255 0.48576 0.95510

24 3.81524 5.08809 4.52154 4.29916 2.99029 4.04872 4.26749 3.85987 4.02104 3.20510 4.47974 4.39720 4.59007 4.39447 4.09969 4.01615 4.13096 3.75712 0.48599 2.97987 30 W - - -

2.68618 4.42225 2.77519 2.73123 3.46354 2.40513 3.72494 3.29354 2.67741 2.69355 4.24690 2.90347 2.73739 3.18146 2.89801 2.37887 2.77519 2.98518 4.58477 3.61503

0.02399 4.13790 4.86025 0.61958 0.77255 0.48576 0.95510

25 2.57936 4.68279 2.82258 2.61918 4.41930 3.19198 3.96650 3.94629 2.85179 3.55307 4.38465 1.60484 3.84154 3.16598 3.26934 1.65488 2.97268 3.46992 5.69604 4.34140 31 n - - -

2.68618 4.42225 2.77519 2.73123 3.46354 2.40513 3.72494 3.29354 2.67741 2.69355 4.24690 2.90347 2.73739 3.18146 2.89801 2.37887 2.77519 2.98518 4.58477 3.61503

0.02399 4.13790 4.86025 0.61958 0.77255 0.48576 0.95510

26 2.30967 5.11996 2.47060 2.32017 4.45306 3.37203 3.65891 3.91137 1.78231 3.43315 4.21896 2.32211 3.83322 2.78572 2.89862 2.68363 2.96093 3.51411 5.59763 4.20656 32 k - - -

2.68618 4.42225 2.77519 2.73123 3.46354 2.40513 3.72494 3.29354 2.67741 2.69355 4.24690 2.90347 2.73739 3.18146 2.89801 2.37887 2.77519 2.98518 4.58477 3.61503

0.02399 4.13790 4.86025 0.61958 0.77255 0.48576 0.95510

27 2.79048 5.05669 3.14690 2.54825 4.39936 3.53509 3.62205 3.80415 1.77404 3.03512 4.12574 3.03822 3.91327 2.51904 1.97408 2.32251 2.99785 3.45314 5.45203 4.16376 33 k - - -

2.68618 4.42225 2.77519 2.73123 3.46354 2.40513 3.72494 3.29354 2.67741 2.69355 4.24690 2.90347 2.73739 3.18146 2.89801 2.37887 2.77519 2.98518 4.58477 3.61503

0.02399 4.13790 4.86025 0.61958 0.77255 0.48576 0.95510

28 2.79337 4.26547 4.15882 3.58692 2.39876 3.88116 3.33782 2.52474 3.45903 1.64474 2.13506 3.81323 4.22121 3.67601 3.64973 3.17215 3.02187 2.43859 4.64173 3.32446 34 l - - -

2.68618 4.42225 2.77519 2.73123 3.46354 2.40513 3.72494 3.29354 2.67741 2.69355 4.24690 2.90347 2.73739 3.18146 2.89801 2.37887 2.77519 2.98518 4.58477 3.61503

0.02399 4.13790 4.86025 0.61958 0.77255 0.48576 0.95510

29 2.42710 4.56146 3.19197 2.63391 3.78008 3.47026 3.72188 3.15628 2.57298 2.39134 3.68239 2.92277 3.87297 2.93541 2.41593 2.48291 2.51142 2.71822 5.13374 3.85472 35 l - - -

2.68618 4.42225 2.77519 2.73123 3.46354 2.40513 3.72494 3.29354 2.67741 2.69355 4.24690 2.90347 2.73739 3.18146 2.89801 2.37887 2.77519 2.98518 4.58477 3.61503

0.02399 4.13790 4.86025 0.61958 0.77255 0.48576 0.95510

30 2.66676 4.91312 2.98865 2.46907 4.25178 3.42949 3.65087 3.67290 1.90504 3.23426 4.03974 2.78469 3.84875 2.78840 2.60950 2.31088 2.20913 3.31756 5.42674 4.09962 36 k - - -

2.68618 4.42225 2.77519 2.73123 3.46354 2.40513 3.72494 3.29354 2.67741 2.69355 4.24690 2.90347 2.73739 3.18146 2.89801 2.37887 2.77519 2.98518 4.58477 3.61503

0.02399 4.13790 4.86025 0.61958 0.77255 0.48576 0.95510

31 2.35589 4.27025 3.52770 3.25506 4.25690 3.07952 4.29431 3.55517 3.24548 3.34570 4.23269 3.41546 3.81686 3.58639 3.52849 2.21406 1.04813 3.10616 5.64278 4.41276 37 t - - -

2.68618 4.42225 2.77519 2.73123 3.46354 2.40513 3.72494 3.29354 2.67741 2.69355 4.24690 2.90347 2.73739 3.18146 2.89801 2.37887 2.77519 2.98518 4.58477 3.61503

0.02399 4.13790 4.86025 0.61958 0.77255 0.48576 0.95510

32 0.90819 4.27705 3.55185 3.28633 4.31751 3.07654 4.33899 3.62102 3.32359 3.38294 4.27421 3.43894 2.90452 3.63134 3.61376 2.55001 2.84657 3.15637 5.68822 4.48700 38 a - - -

2.68618 4.42225 2.77519 2.73123 3.46354 2.40513 3.72494 3.29354 2.67741 2.69355 4.24690 2.90347 2.73739 3.18146 2.89801 2.37887 2.77519 2.98518 4.58477 3.61503

0.02399 4.13790 4.86025 0.61958 0.77255 0.48576 0.95510

33 3.02976 4.77030 3.75720 3.69437 4.81559 0.41625 4.79027 4.48786 3.91128 4.10567 5.08944 3.92261 4.16926 4.21493 4.11040 3.21057 3.53155 3.96477 5.83234 4.92928 39 G - - -

2.68618 4.42225 2.77519 2.73123 3.46354 2.40513 3.72494 3.29354 2.67741 2.69355 4.24690 2.90347 2.73739 3.18146 2.89801 2.37887 2.77519 2.98518 4.58477 3.61503

0.02399 4.13790 4.86025 0.61958 0.77255 0.48576 0.95510

34 2.64158 4.68109 3.15758 2.58423 3.52846 3.49646 3.67731 3.27798 2.30186 2.49037 3.76139 3.07043 3.87917 2.47307 2.23346 2.72091 2.86728 2.99702 5.17678 3.32853 40 r - - -

2.68618 4.42225 2.77519 2.73123 3.46354 2.40513 3.72494 3.29354 2.67741 2.69355 4.24690 2.90347 2.73739 3.18146 2.89801 2.37887 2.77519 2.98518 4.58477 3.61503

0.02399 4.13790 4.86025 0.61958 0.77255 0.48576 0.95510

35 1.82452 2.01309 4.03800 3.55981 3.70872 3.27823 4.28487 2.77668 3.46022 2.73091 3.66914 3.63276 3.92215 3.71388 3.68844 2.68170 2.26247 2.23335 5.20872 4.02108 41 a - - -

2.68618 4.42225 2.77519 2.73123 3.46354 2.40513 3.72494 3.29354 2.67741 2.69355 4.24690 2.90347 2.73739 3.18146 2.89801 2.37887 2.77519 2.98518 4.58477 3.61503

0.02399 4.13790 4.86025 0.61958 0.77255 0.48576 0.95510

36 2.59229 4.52863 3.19892 2.63722 3.70410 3.49227 3.37816 2.94801 2.60745 2.75847 3.32722 2.62070 3.87761 2.94400 2.79244 2.42606 2.70441 2.42698 5.07786 3.50093 42 s - - -

2.68618 4.42225 2.77519 2.73123 3.46354 2.40513 3.72494 3.29354 2.67741 2.69355 4.24690 2.90347 2.73739 3.18146 2.89801 2.37887 2.77519 2.98518 4.58477 3.61503

0.02399 4.13790 4.86025 0.61958 0.77255 0.48576 0.95510

37 2.58974 4.33864 3.42254 2.86172 3.47561 3.55194 3.80796 2.62804 2.81136 2.14615 3.44600 2.79655 3.93750 3.12575 3.17175 2.49938 2.46003 2.60946 4.91546 3.11621 43 l - - -

2.68618 4.42225 2.77519 2.73123 3.46354 2.40513 3.72494 3.29354 2.67741 2.69355 4.24690 2.90347 2.73739 3.18146 2.89801 2.37887 2.77519 2.98518 4.58477 3.61503

0.02399 4.13790 4.86025 0.61958 0.77255 0.48576 0.95510

38 2.69025 5.04837 2.92236 2.25088 4.36682 2.70116 3.18874 3.80556 1.80922 3.32582 4.11111 2.92817 3.83908 2.73180 2.56414 2.67460 2.68044 3.42801 5.48419 4.13323 44 k - - -

2.68618 4.42225 2.77519 2.73123 3.46354 2.40513 3.72494 3.29354 2.67741 2.69355 4.24690 2.90347 2.73739 3.18146 2.89801 2.37887 2.77519 2.98518 4.58477 3.61503

0.02399 4.13790 4.86025 0.61958 0.77255 0.48576 0.95510

39 2.68078 5.02868 2.98048 2.31079 4.34598 3.44910 3.60238 3.78085 2.06622 3.08128 4.08726 2.94107 3.33046 2.51047 2.11246 2.39108 2.90303 3.40660 5.46080 4.11664 45 k - - -

2.68618 4.42225 2.77519 2.73123 3.46354 2.40513 3.72494 3.29354 2.67741 2.69355 4.24690 2.90347 2.73739 3.18146 2.89801 2.37887 2.77519 2.98518 4.58477 3.61503

0.02399 4.13790 4.86025 0.61958 0.77255 0.48576 0.95510

40 2.45886 4.93554 2.92406 2.19075 4.21482 3.40821 3.60494 3.65328 2.16264 3.04613 3.59803 2.50631 3.80277 2.61430 2.83232 2.40520 2.72679 3.29073 5.40942 4.04724 46 k - - -

2.68618 4.42225 2.77519 2.73123 3.46354 2.40513 3.72494 3.29354 2.67741 2.69355 4.24690 2.90347 2.73739 3.18146 2.89801 2.37887 2.77519 2.98518 4.58477 3.61503

0.02399 4.13790 4.86025 0.61958 0.77255 0.48576 0.95510

41 2.60467 4.65326 3.11173 2.54950 3.85548 3.12884 3.67683 3.25044 2.41542 2.55758 3.45385 2.42502 3.85555 2.86494 2.57711 2.55111 2.83496 2.96591 4.20061 3.88118 47 k - - -

2.68618 4.42225 2.77519 2.73123 3.46354 2.40513 3.72494 3.29354 2.67741 2.69355 4.24690 2.90347 2.73739 3.18146 2.89801 2.37887 2.77519 2.98518 4.58477 3.61503

0.02399 4.13790 4.86025 0.61958 0.77255 0.48576 0.95510

42 2.51538 4.99423 2.64316 2.34387 4.28673 2.88347 3.60976 3.73691 2.13672 3.27939 4.05825 2.55270 3.02896 2.73155 2.86344 2.61426 2.86061 3.35842 5.46535 3.38589 48 k - - -

2.68618 4.42225 2.77519 2.73123 3.46354 2.40513 3.72494 3.29354 2.67741 2.69355 4.24690 2.90347 2.73739 3.18146 2.89801 2.37887 2.77519 2.98518 4.58477 3.61503

0.02399 4.13790 4.86025 0.61958 0.77255 0.48576 0.95510

43 2.59468 4.77355 2.72872 2.45403 3.49015 3.08700 3.64705 3.43808 2.45621 3.04770 3.86432 2.96925 3.08012 2.66454 2.91753 2.33695 2.48266 2.95615 5.29747 3.96664 49 s - - -

2.68618 4.42225 2.77519 2.73123 3.46354 2.40513 3.72494 3.29354 2.67741 2.69355 4.24690 2.90347 2.73739 3.18146 2.89801 2.37887 2.77519 2.98518 4.58477 3.61503

0.02399 4.13790 4.86025 0.61958 0.77255 0.48576 0.95510

44 2.42588 4.95429 2.90390 2.29911 4.25418 2.83776 3.61309 3.69543 2.19783 3.24588 4.03064 2.69606 3.80662 2.73805 2.57393 2.45253 2.48838 3.32526 5.43702 4.07335 50 k - - -

2.68623 4.42230 2.77525 2.73118 3.46359 2.40497 3.72500 3.29359 2.67746 2.69345 4.24695 2.90352 2.73729 3.18152 2.89806 2.37883 2.77525 2.98524 4.58482 3.61508

0.59584 1.53188 1.45771 0.52216 0.89953 0.48576 0.95510

45 2.50909 4.67215 3.15986 2.57117 3.90629 3.46094 3.61070 3.29423 1.95994 2.91455 3.76692 3.03949 3.84490 2.79102 2.28700 2.71089 2.65618 3.01000 4.01802 3.89153 54 k - - -

2.68618 4.42225 2.77519 2.73123 3.46354 2.40513 3.72494 3.29354 2.67741 2.69355 4.24690 2.90347 2.73739 3.18146 2.89801 2.37887 2.77519 2.98518 4.58477 3.61503

0.03107 3.88298 4.60533 0.61958 0.77255 0.61460 0.77839

46 2.45676 4.90211 2.99263 2.33333 4.19197 3.42834 3.26919 3.60631 2.00080 3.16130 3.96987 2.93930 3.81623 2.58455 2.29041 2.66110 2.87118 2.99718 5.34820 4.03115 55 k - - -

2.68618 4.42225 2.77519 2.73123 3.46354 2.40513 3.72494 3.29354 2.67741 2.69355 4.24690 2.90347 2.73739 3.18146 2.89801 2.37887 2.77519 2.98518 4.58477 3.61503

0.02883 3.95665 4.67899 0.61958 0.77255 0.66591 0.72115

47 2.59810 4.47331 3.27755 2.71248 3.60781 3.48602 3.70849 3.00528 2.53087 2.68952 3.58467 3.15258 3.88322 2.95987 2.24432 2.74241 2.54657 2.52813 5.00009 2.94505 56 r - - -

2.68618 4.42225 2.77519 2.73123 3.46354 2.40513 3.72494 3.29354 2.67741 2.69355 4.24690 2.90347 2.73739 3.18146 2.89801 2.37887 2.77519 2.98518 4.58477 3.61503

0.02883 3.95665 4.67899 0.61958 0.77255 0.46298 0.99265

48 2.57646 3.75697 3.62032 3.04864 2.91441 3.59472 3.86440 2.68603 2.98198 2.18058 3.31659 3.40430 3.41004 3.26565 2.97702 2.54946 2.66872 2.47342 4.79774 2.80952 57 l - - -

2.68618 4.42225 2.77519 2.73123 3.46354 2.40513 3.72494 3.29354 2.67741 2.69355 4.24690 2.90347 2.73739 3.18146 2.89801 2.37887 2.77519 2.98518 4.58477 3.61503

0.02436 4.12283 4.84518 0.61958 0.77255 0.54306 0.86980

49 0.93623 4.20022 3.60199 3.35617 4.35087 3.00141 4.38203 3.67457 3.39787 3.45392 4.31532 3.43929 3.77184 3.68621 3.66983 2.05636 2.78534 3.15819 5.73027 4.52390 58 a - - -

2.68618 4.42225 2.77519 2.73123 3.46354 2.40513 3.72494 3.29354 2.67741 2.69355 4.24690 2.90347 2.73739 3.18146 2.89801 2.37887 2.77519 2.98518 4.58477 3.61503

0.02436 4.12283 4.84518 0.61958 0.77255 0.54306 0.86980

50 2.61586 4.64818 3.13624 2.57034 3.35951 3.47880 3.67745 3.24156 2.17174 2.88559 3.73330 3.05915 3.86463 2.87130 2.27435 2.47703 2.84483 2.79365 5.16007 3.29154 59 k - - -

2.68618 4.42225 2.77519 2.73123 3.46354 2.40513 3.72494 3.29354 2.67741 2.69355 4.24690 2.90347 2.73739 3.18146 2.89801 2.37887 2.77519 2.98518 4.58477 3.61503

0.02436 4.12283 4.84518 0.61958 0.77255 0.45153 1.01240

51 3.25285 4.52457 5.04838 4.53364 3.56193 4.67262 5.18924 1.01917 4.41980 1.83997 3.34884 4.74666 4.91779 4.63619 4.58491 4.05697 3.50397 1.49544 5.59170 4.42311 60 i - - -

2.68618 4.42225 2.77519 2.73123 3.46354 2.40513 3.72494 3.29354 2.67741 2.69355 4.24690 2.90347 2.73739 3.18146 2.89801 2.37887 2.77519 2.98518 4.58477 3.61503

0.02318 4.17189 4.89423 0.61958 0.77255 0.48576 0.95510

52 2.73904 5.15010 2.43338 1.48949 4.43709 3.38064 3.67521 3.30593 2.48267 3.42730 4.22138 2.86049 3.84595 2.50182 2.97472 2.70309 2.98211 3.50750 5.60874 4.20782 61 e - - -

2.68618 4.42225 2.77519 2.73123 3.46354 2.40513 3.72494 3.29354 2.67741 2.69355 4.24690 2.90347 2.73739 3.18146 2.89801 2.37887 2.77519 2.98518 4.58477 3.61503

0.02318 4.17189 4.89423 0.61958 0.77255 0.48576 0.95510

53 3.45702 4.75352 5.08405 4.51656 3.13846 4.74894 5.04411 1.93912 4.36641 0.79792 2.54012 4.77791 4.88609 4.43984 4.46082 4.10659 3.67335 2.32294 5.27712 4.24281 62 l - - -

2.68618 4.42225 2.77519 2.73123 3.46354 2.40513 3.72494 3.29354 2.67741 2.69355 4.24690 2.90347 2.73739 3.18146 2.89801 2.37887 2.77519 2.98518 4.58477 3.61503

0.02318 4.17189 4.89423 0.61958 0.77255 0.48576 0.95510

54 2.37638 4.33104 3.40460 3.22232 4.52348 2.62816 4.37518 4.02101 3.36772 3.69483 4.52368 3.40037 3.81883 3.65521 3.67207 0.84683 2.88074 3.41620 5.84052 4.61317 63 s - - -

2.68618 4.42225 2.77519 2.73123 3.46354 2.40513 3.72494 3.29354 2.67741 2.69355 4.24690 2.90347 2.73739 3.18146 2.89801 2.37887 2.77519 2.98518 4.58477 3.61503

0.02318 4.17189 4.89423 0.61958 0.77255 0.48576 0.95510

55 2.61711 4.81891 2.59651 2.27342 4.06697 3.42962 3.65333 3.47248 2.45813 2.77241 3.90037 2.96485 3.05271 2.80465 2.92231 2.64992 2.29305 2.83692 5.33181 3.99476 64 e - - -

2.68618 4.42225 2.77519 2.73123 3.46354 2.40513 3.72494 3.29354 2.67741 2.69355 4.24690 2.90347 2.73739 3.18146 2.89801 2.37887 2.77519 2.98518 4.58477 3.61503

0.02318 4.17189 4.89423 0.61958 0.77255 0.48576 0.95510

56 2.83796 4.92173 3.32626 2.70874 4.19633 3.60762 3.69216 3.52991 1.34942 2.77866 4.00327 3.16336 3.98537 2.84909 2.34699 2.88492 3.04609 2.87499 5.35818 4.10405 65 k - - -

2.68618 4.42225 2.77519 2.73123 3.46354 2.40513 3.72494 3.29354 2.67741 2.69355 4.24690 2.90347 2.73739 3.18146 2.89801 2.37887 2.77519 2.98518 4.58477 3.61503

0.02318 4.17189 4.89423 0.61958 0.77255 0.48576 0.95510

57 3.15280 4.51149 4.76244 4.28035 3.53280 4.39854 4.95372 1.91248 4.12387 1.73964 3.38762 4.49314 4.74779 4.40591 4.31264 3.79633 3.42819 0.95903 5.47564 4.25093 66 v - - -

2.68618 4.42225 2.77519 2.73123 3.46354 2.40513 3.72494 3.29354 2.67741 2.69355 4.24690 2.90347 2.73739 3.18146 2.89801 2.37887 2.77519 2.98518 4.58477 3.61503

0.02318 4.17189 4.89423 0.61958 0.77255 0.48576 0.95510

58 2.94715 2.49060 4.72953 4.16932 3.42049 4.22221 4.64669 1.56729 4.03684 1.82177 3.30688 4.31061 4.54552 4.23192 4.16425 3.55866 3.19230 1.55669 5.16623 3.98627 67 v - - -

2.68618 4.42225 2.77519 2.73123 3.46354 2.40513 3.72494 3.29354 2.67741 2.69355 4.24690 2.90347 2.73739 3.18146 2.89801 2.37887 2.77519 2.98518 4.58477 3.61503

0.02318 4.17189 4.89423 0.61958 0.77255 0.48576 0.95510

59 2.62705 4.81940 1.80329 2.46730 4.23844 3.34324 3.76012 3.61208 2.60460 3.25673 4.07911 2.97545 3.84253 2.91894 3.07622 2.44279 2.28685 2.81713 5.50130 4.15292 68 d - - -

2.68618 4.42225 2.77519 2.73123 3.46354 2.40513 3.72494 3.29354 2.67741 2.69355 4.24690 2.90347 2.73739 3.18146 2.89801 2.37887 2.77519 2.98518 4.58477 3.61503

0.02318 4.17189 4.89423 0.61958 0.77255 0.48576 0.95510

60 2.54828 4.65460 2.58565 2.60762 4.30563 3.24419 3.89245 3.72085 2.77195 3.35247 4.17397 3.06055 3.83111 3.07361 3.21903 1.56259 2.00823 3.30442 5.58500 4.26219 69 s - - -

2.68618 4.42225 2.77519 2.73123 3.46354 2.40513 3.72494 3.29354 2.67741 2.69355 4.24690 2.90347 2.73739 3.18146 2.89801 2.37887 2.77519 2.98518 4.58477 3.61503

0.02318 4.17189 4.89423 0.61958 0.77255 0.48576 0.95510

61 2.06191 4.66993 3.10943 2.31138 3.88865 3.47529 3.70046 3.24382 2.25528 2.69384 3.76533 3.05840 3.87128 2.88745 2.95011 2.70224 2.72463 2.58990 5.21345 3.91482 70 a - - -

2.68618 4.42225 2.77519 2.73123 3.46354 2.40513 3.72494 3.29354 2.67741 2.69355 4.24690 2.90347 2.73739 3.18146 2.89801 2.37887 2.77519 2.98518 4.58477 3.61503

0.02318 4.17189 4.89423 0.61958 0.77255 0.48576 0.95510

62 2.92608 5.46832 1.70523 1.38138 4.77632 3.32070 3.78978 4.26866 2.73378 3.77386 4.59278 2.78516 3.89671 2.93934 3.29874 2.83359 2.75345 3.84376 5.93749 4.45873 71 e - - -

2.68618 4.42225 2.77519 2.73123 3.46354 2.40513 3.72494 3.29354 2.67741 2.69355 4.24690 2.90347 2.73739 3.18146 2.89801 2.37887 2.77519 2.98518 4.58477 3.61503

0.02318 4.17189 4.89423 0.61958 0.77255 0.48576 0.95510

63 3.03676 5.17162 3.48389 2.81706 4.62275 3.69738 3.67774 3.96255 1.67258 3.43274 4.28821 3.23322 4.06921 2.81119 1.29626 3.04051 2.78908 3.63696 5.50869 4.32093 72 r - - -

2.68618 4.42225 2.77519 2.73123 3.46354 2.40513 3.72494 3.29354 2.67741 2.69355 4.24690 2.90347 2.73739 3.18146 2.89801 2.37887 2.77519 2.98518 4.58477 3.61503

0.02318 4.17189 4.89423 0.61958 0.77255 0.48576 0.95510

64 3.33984 4.65655 4.96779 4.42315 3.25313 4.62909 5.00432 1.94887 4.27860 0.89434 3.04497 4.66966 4.83793 4.42212 4.41149 3.99323 3.57538 1.82582 5.33359 4.24016 73 l - - -

2.68618 4.42225 2.77519 2.73123 3.46354 2.40513 3.72494 3.29354 2.67741 2.69355 4.24690 2.90347 2.73739 3.18146 2.89801 2.37887 2.77519 2.98518 4.58477 3.61503

0.02318 4.17189 4.89423 0.61958 0.77255 0.48576 0.95510

65 2.52875 4.98015 3.07442 2.27809 4.27391 3.50499 3.64090 3.68642 2.12275 3.23684 4.05004 3.01665 3.89301 2.77680 1.76690 2.74838 2.95626 3.34801 5.41485 3.35533 74 r - - -

2.68618 4.42225 2.77519 2.73123 3.46354 2.40513 3.72494 3.29354 2.67741 2.69355 4.24690 2.90347 2.73739 3.18146 2.89801 2.37887 2.77519 2.98518 4.58477 3.61503

0.02318 4.17189 4.89423 0.61958 0.77255 0.48576 0.95510

66 2.47313 3.97696 2.07795 2.40134 4.32553 3.34486 3.70452 3.76844 2.51938 3.33607 4.12938 2.02348 3.82217 2.84317 3.00242 2.49653 2.90348 3.37751 5.53556 4.16552 75 n - - -

2.68618 4.42225 2.77519 2.73123 3.46354 2.40513 3.72494 3.29354 2.67741 2.69355 4.24690 2.90347 2.73739 3.18146 2.89801 2.37887 2.77519 2.98518 4.58477 3.61503

0.02318 4.17189 4.89423 0.61958 0.77255 0.48576 0.95510

67 2.50701 4.31821 3.81705 3.44400 3.96785 3.30795 4.36950 2.86724 3.33996 2.85653 3.91418 3.60662 3.98447 3.69239 3.60899 2.74534 1.10191 2.29028 5.50959 4.27805 76 t - - -

2.68618 4.42225 2.77519 2.73123 3.46354 2.40513 3.72494 3.29354 2.67741 2.69355 4.24690 2.90347 2.73739 3.18146 2.89801 2.37887 2.77519 2.98518 4.58477 3.61503

0.02318 4.17189 4.89423 0.61958 0.77255 0.48576 0.95510

68 3.50942 4.88889 4.66973 4.32135 3.27856 4.35914 4.88456 2.50874 4.08677 0.57799 3.22623 4.56823 4.74956 4.38185 4.20951 3.98149 3.79359 2.62188 5.25526 4.04426 77 L - - -

2.68618 4.42225 2.77519 2.73123 3.46354 2.40513 3.72494 3.29354 2.67741 2.69355 4.24690 2.90347 2.73739 3.18146 2.89801 2.37887 2.77519 2.98518 4.58477 3.61503

0.02318 4.17189 4.89423 0.61958 0.77255 0.48576 0.95510

69 1.98114 3.10437 4.18496 3.62442 3.37899 3.78892 4.23578 1.71855 3.51221 2.19919 3.33655 3.83130 4.20052 3.75454 3.71679 3.10779 2.76985 2.06869 4.95216 3.75565 78 i - - -

2.68618 4.42225 2.77519 2.73123 3.46354 2.40513 3.72494 3.29354 2.67741 2.69355 4.24690 2.90347 2.73739 3.18146 2.89801 2.37887 2.77519 2.98518 4.58477 3.61503

0.02318 4.17189 4.89423 0.61958 0.77255 0.48576 0.95510

70 3.38191 5.12656 3.47442 3.27459 3.54089 3.74007 0.64247 4.13248 3.11167 3.58329 4.65814 3.66755 4.31394 3.64776 3.35392 3.45307 3.69989 3.86219 4.99896 3.48545 79 H - - -

2.68618 4.42225 2.77519 2.73123 3.46354 2.40513 3.72494 3.29354 2.67741 2.69355 4.24690 2.90347 2.73739 3.18146 2.89801 2.37887 2.77519 2.98518 4.58477 3.61503

0.02318 4.17189 4.89423 0.61958 0.77255 0.48576 0.95510

71 3.24719 5.38720 2.66025 0.66358 4.77676 3.49454 4.12204 4.29308 3.04373 3.86623 4.83885 3.14955 4.11689 3.33886 3.47555 3.20540 3.55751 3.95009 5.89065 4.62790 80 E - - -

2.68618 4.42225 2.77519 2.73123 3.46354 2.40513 3.72494 3.29354 2.67741 2.69355 4.24690 2.90347 2.73739 3.18146 2.89801 2.37887 2.77519 2.98518 4.58477 3.61503

0.02318 4.17189 4.89423 0.61958 0.77255 0.48576 0.95510

72 3.27400 4.60710 4.89200 4.31931 3.17469 4.53161 4.85228 2.06984 4.19016 1.23122 1.69113 4.55713 4.73731 4.29948 4.30810 3.86831 3.49682 2.00652 5.20352 4.13893 81 l - - -

2.68618 4.42225 2.77519 2.73123 3.46354 2.40513 3.72494 3.29354 2.67741 2.69355 4.24690 2.90347 2.73739 3.18146 2.89801 2.37887 2.77519 2.98518 4.58477 3.61503

0.02318 4.17189 4.89423 0.61958 0.77255 0.48576 0.95510

73 2.97767 0.49333 4.54946 4.36497 4.30151 3.52776 4.94768 3.50716 4.19852 3.40312 4.53767 4.27439 4.25660 4.53252 4.26575 3.24836 3.47023 3.22830 5.64053 4.58398 82 C - - -

2.68618 4.42225 2.77519 2.73123 3.46354 2.40513 3.72494 3.29354 2.67741 2.69355 4.24690 2.90347 2.73739 3.18146 2.89801 2.37887 2.77519 2.98518 4.58477 3.61503

0.02318 4.17189 4.89423 0.61958 0.77255 0.48576 0.95510

74 3.38191 5.12656 3.47442 3.27459 3.54089 3.74007 0.64247 4.13248 3.11167 3.58329 4.65814 3.66755 4.31394 3.64776 3.35392 3.45307 3.69989 3.86219 4.99896 3.48545 83 H - - -

2.68618 4.42225 2.77519 2.73123 3.46354 2.40513 3.72494 3.29354 2.67741 2.69355 4.24690 2.90347 2.73739 3.18146 2.89801 2.37887 2.77519 2.98518 4.58477 3.61503

0.02318 4.17189 4.89423 0.61958 0.77255 0.48576 0.95510

75 1.52285 4.34248 3.97730 3.46913 3.57912 3.69369 4.30338 2.37653 3.37339 1.96651 3.48901 3.74165 4.18593 3.67744 3.65469 3.04480 3.02701 1.92940 5.21557 4.00145 84 a - - -

2.68618 4.42225 2.77519 2.73123 3.46354 2.40513 3.72494 3.29354 2.67741 2.69355 4.24690 2.90347 2.73739 3.18146 2.89801 2.37887 2.77519 2.98518 4.58477 3.61503

0.02318 4.17189 4.89423 0.61958 0.77255 0.48576 0.95510

76 0.82846 4.22865 3.66022 3.43736 4.39194 3.03199 4.45240 3.69090 3.47793 3.49581 4.37020 3.49620 3.81090 3.76750 3.73474 2.24283 2.82658 3.17956 5.78100 4.57582 85 a - - -

2.68618 4.42225 2.77519 2.73123 3.46354 2.40513 3.72494 3.29354 2.67741 2.69355 4.24690 2.90347 2.73739 3.18146 2.89801 2.37887 2.77519 2.98518 4.58477 3.61503

0.02318 4.17189 4.89423 0.61958 0.77255 0.48576 0.95510

77 2.07111 2.93417 3.51985 2.98308 3.67283 3.37421 3.93586 3.02945 2.93452 2.74511 3.62393 3.32586 3.88900 2.96598 3.28683 2.24965 2.15272 2.59120 5.09587 3.86329 86 a - - -

2.68618 4.42225 2.77519 2.73123 3.46354 2.40513 3.72494 3.29354 2.67741 2.69355 4.24690 2.90347 2.73739 3.18146 2.89801 2.37887 2.77519 2.98518 4.58477 3.61503

0.02318 4.17189 4.89423 0.61958 0.77255 0.48576 0.95510

78 3.88223 5.14079 4.57996 4.36763 3.03965 4.09260 4.32068 3.93148 4.09071 3.26970 4.54920 4.45978 4.63710 4.46211 4.16246 4.08278 4.20019 3.82946 0.45023 3.02785 87 W - - -

2.68618 4.42225 2.77519 2.73123 3.46354 2.40513 3.72494 3.29354 2.67741 2.69355 4.24690 2.90347 2.73739 3.18146 2.89801 2.37887 2.77519 2.98518 4.58477 3.61503

0.02318 4.17189 4.89423 0.61958 0.77255 0.48576 0.95510

79 2.64970 4.37857 4.60667 4.03312 3.25848 4.22075 4.56674 1.94737 3.91210 1.33169 2.46371 4.24138 4.51287 4.08579 4.06089 3.53869 3.23412 1.83009 5.08067 3.94776 88 l - - -

2.68618 4.42225 2.77519 2.73123 3.46354 2.40513 3.72494 3.29354 2.67741 2.69355 4.24690 2.90347 2.73739 3.18146 2.89801 2.37887 2.77519 2.98518 4.58477 3.61503

0.02318 4.17189 4.89423 0.61958 0.77255 0.48576 0.95510

80 3.20074 4.50429 4.96919 4.40801 2.55136 4.50787 4.84662 1.31769 4.28352 1.47565 3.08631 4.57911 4.73925 4.39177 4.37718 3.85356 3.43113 1.85910 5.19311 4.04505 89 i - - -

2.68618 4.42225 2.77519 2.73123 3.46354 2.40513 3.72494 3.29354 2.67741 2.69355 4.24690 2.90347 2.73739 3.18146 2.89801 2.37887 2.77519 2.98518 4.58477 3.61503

0.02318 4.17189 4.89423 0.61958 0.77255 0.48576 0.95510

81 2.95260 5.45642 1.24298 2.21087 4.82766 3.29252 3.84739 4.35851 2.84903 3.86231 4.69814 2.10080 3.91046 3.01160 3.43350 2.49750 3.25393 3.91362 6.01656 4.52132 90 d - - -

2.68618 4.42225 2.77519 2.73123 3.46354 2.40513 3.72494 3.29354 2.67741 2.69355 4.24690 2.90347 2.73739 3.18146 2.89801 2.37887 2.77519 2.98518 4.58477 3.61503

0.02318 4.17189 4.89423 0.61958 0.77255 0.48576 0.95510

82 2.72066 5.07317 2.89124 2.41049 4.39465 2.29227 3.17468 3.83649 2.07556 3.35726 4.14742 2.36005 3.85867 2.76138 2.51777 2.70346 2.94939 3.45932 5.51434 4.16244 91 k - - -

2.68618 4.42225 2.77519 2.73123 3.46354 2.40513 3.72494 3.29354 2.67741 2.69355 4.24690 2.90347 2.73739 3.18146 2.89801 2.37887 2.77519 2.98518 4.58477 3.61503

0.05905 4.17189 3.17217 0.61958 0.77255 0.48576 0.95510

83 2.60891 4.35708 3.43525 2.33034 3.50973 3.57868 3.83305 2.50214 2.82584 2.38693 3.21963 3.29310 3.95929 3.14011 3.19259 2.61295 2.67475 2.14191 4.95798 3.71640 92 v - - -

2.68618 4.42225 2.77519 2.73123 3.46354 2.40513 3.72494 3.29354 2.67741 2.69355 4.24690 2.90347 2.73739 3.18146 2.89801 2.37887 2.77519 2.98518 4.58477 3.61503

0.25673 4.13686 1.55852 0.61958 0.77255 0.52729 0.89209

84 2.30745 4.37398 3.26804 2.72153 3.46328 3.47864 3.23497 2.90132 2.65518 2.59151 2.74858 2.94980 3.86888 3.00060 3.02660 2.72663 2.79006 2.66238 4.89095 3.15341 93 a - - -

2.68618 4.42225 2.77519 2.73123 3.46354 2.40513 3.72494 3.29354 2.67741 2.69355 4.24690 2.90347 2.73739 3.18146 2.89801 2.37887 2.77519 2.98518 4.58477 3.61503

0.03021 3.91035 4.63269 0.61958 0.77255 0.35637 1.20469

85 2.73582 5.14126 2.98365 2.31726 4.49758 3.47320 3.27191 3.93951 1.77864 3.41805 4.19309 2.77340 3.86367 2.31741 2.30416 2.50281 2.95326 3.54119 5.53598 4.19212 94 k - - -

2.68618 4.42225 2.77519 2.73123 3.46354 2.40513 3.72494 3.29354 2.67741 2.69355 4.24690 2.90347 2.73739 3.18146 2.89801 2.37887 2.77519 2.98518 4.58477 3.61503

0.02318 4.17189 4.89423 0.61958 0.77255 0.48576 0.95510

86 2.74973 5.23937 2.23638 2.11748 4.55258 2.86839 3.67335 4.03505 2.31184 3.53418 4.30981 2.41732 2.40545 2.79666 3.01039 2.69563 2.99649 3.61698 5.68935 4.26236 95 e - - -

2.68618 4.42225 2.77519 2.73123 3.46354 2.40513 3.72494 3.29354 2.67741 2.69355 4.24690 2.90347 2.73739 3.18146 2.89801 2.37887 2.77519 2.98518 4.58477 3.61503

0.02318 4.17189 4.89423 0.61958 0.77255 0.48576 0.95510

87 2.31854 4.66386 2.99750 2.59510 4.26670 2.25427 3.83431 3.69723 2.67009 3.30090 4.11189 3.05299 2.23373 2.67443 3.10958 2.31106 2.87340 3.28803 5.52695 4.20875 96 p - - -

2.68618 4.42225 2.77519 2.73123 3.46354 2.40513 3.72494 3.29354 2.67741 2.69355 4.24690 2.90347 2.73739 3.18146 2.89801 2.37887 2.77519 2.98518 4.58477 3.61503

0.02318 4.17189 4.89423 0.61958 0.77255 0.48576 0.95510

88 3.38191 5.12656 3.47442 3.27459 3.54089 3.74007 0.64247 4.13248 3.11167 3.58329 4.65814 3.66755 4.31394 3.64776 3.35392 3.45307 3.69989 3.86219 4.99896 3.48545 97 H - - -

2.68618 4.42225 2.77519 2.73123 3.46354 2.40513 3.72494 3.29354 2.67741 2.69355 4.24690 2.90347 2.73739 3.18146 2.89801 2.37887 2.77519 2.98518 4.58477 3.61503

0.02318 4.17189 4.89423 0.61958 0.77255 0.48576 0.95510

89 3.08163 4.81638 3.81580 3.75833 4.87434 0.38747 4.84968 4.55733 3.97972 4.16979 5.15549 3.98147 4.21612 4.28075 4.17281 3.26377 3.58596 4.02759 5.87966 4.98999 98 G - - -

2.68618 4.42225 2.77519 2.73123 3.46354 2.40513 3.72494 3.29354 2.67741 2.69355 4.24690 2.90347 2.73739 3.18146 2.89801 2.37887 2.77519 2.98518 4.58477 3.61503

0.02318 4.17189 4.89423 0.61958 0.77255 0.48576 0.95510

90 2.31095 4.85252 2.66698 2.43289 4.16364 3.40021 3.65361 3.59045 2.43916 3.17429 3.97457 2.95211 2.60504 2.79356 2.51757 2.45979 2.85152 2.95001 5.39512 4.04784 99 a - - -

2.68618 4.42225 2.77519 2.73123 3.46354 2.40513 3.72494 3.29354 2.67741 2.69355 4.24690 2.90347 2.73739 3.18146 2.89801 2.37887 2.77519 2.98518 4.58477 3.61503

0.02318 4.17189 4.89423 0.61958 0.77255 0.48576 0.95510

91 2.10417 3.47482 3.59303 2.77640 3.00706 3.19689 3.86898 2.72204 2.96316 2.45109 3.14639 3.39150 3.97717 3.25120 3.08288 2.85303 2.82277 2.20158 4.83773 3.61614 100 a - - -

2.68618 4.42225 2.77519 2.73123 3.46354 2.40513 3.72494 3.29354 2.67741 2.69355 4.24690 2.90347 2.73739 3.18146 2.89801 2.37887 2.77519 2.98518 4.58477 3.61503

0.02318 4.17189 4.89423 0.61958 0.77255 0.48576 0.95510

92 3.74178 4.95125 4.85053 4.54311 1.19268 4.50151 3.61463 3.36654 4.34471 2.65592 3.94120 4.23757 4.80995 4.29219 4.33518 3.89592 3.96507 3.31944 1.53633 2.02359 101 f - - -

2.68618 4.42225 2.77519 2.73123 3.46354 2.40513 3.72494 3.29354 2.67741 2.69355 4.24690 2.90347 2.73739 3.18146 2.89801 2.37887 2.77519 2.98518 4.58477 3.61503

0.02318 4.17189 4.89423 0.61958 0.77255 0.48576 0.95510

93 2.86226 5.08491 3.25062 2.40580 4.42197 3.59528 3.64170 3.82149 1.36499 3.32791 4.15205 3.10033 3.96449 2.77264 2.11681 2.86289 3.05933 3.48456 4.11248 4.18667 102 k - - -

2.68618 4.42225 2.77519 2.73123 3.46354 2.40513 3.72494 3.29354 2.67741 2.69355 4.24690 2.90347 2.73739 3.18146 2.89801 2.37887 2.77519 2.98518 4.58477 3.61503

0.02318 4.17189 4.89423 0.61958 0.77255 0.48576 0.95510

94 2.26998 4.39201 3.37102 2.80489 2.99760 3.28618 3.78752 2.90780 2.60846 2.51796 3.22364 3.23826 3.92942 3.07867 2.53572 2.78512 2.83048 2.67240 4.95952 2.99797 103 a - - -

2.68618 4.42225 2.77519 2.73123 3.46354 2.40513 3.72494 3.29354 2.67741 2.69355 4.24690 2.90347 2.73739 3.18146 2.89801 2.37887 2.77519 2.98518 4.58477 3.61503

0.02318 4.17189 4.89423 0.61958 0.77255 0.48576 0.95510

95 3.73209 4.99352 4.65073 4.31846 2.05576 4.43274 3.56535 3.55481 4.06786 2.89605 4.12656 4.12635 4.76169 4.15693 4.13305 3.82727 3.95517 3.44796 1.15673 1.52544 104 w - - -

2.68618 4.42225 2.77519 2.73123 3.46354 2.40513 3.72494 3.29354 2.67741 2.69355 4.24690 2.90347 2.73739 3.18146 2.89801 2.37887 2.77519 2.98518 4.58477 3.61503

0.02318 4.17189 4.89423 0.61958 0.77255 0.48576 0.95510

96 1.27851 4.44030 3.31500 2.91893 4.22047 2.35523 4.02842 3.61614 2.86938 3.27864 4.11825 3.25237 3.83695 3.24991 2.73152 2.60111 2.85934 3.19354 5.53033 4.27855 105 a - - -

2.68618 4.42225 2.77519 2.73123 3.46354 2.40513 3.72494 3.29354 2.67741 2.69355 4.24690 2.90347 2.73739 3.18146 2.89801 2.37887 2.77519 2.98518 4.58477 3.61503

0.02318 4.17189 4.89423 0.61958 0.77255 0.48576 0.95510

97 2.14041 5.04544 3.05397 2.50502 4.38213 3.50117 3.63903 3.79768 2.06231 3.32057 4.12506 2.68473 3.89660 2.60199 1.91480 2.75616 2.97870 3.43963 5.47273 4.16217 106 r - - -

2.68618 4.42225 2.77519 2.73123 3.46354 2.40513 3.72494 3.29354 2.67741 2.69355 4.24690 2.90347 2.73739 3.18146 2.89801 2.37887 2.77519 2.98518 4.58477 3.61503

0.02318 4.17189 4.89423 0.61958 0.77255 0.48576 0.95510

98 2.53208 4.88977 3.22120 2.61499 4.16584 3.55270 3.66548 3.10234 1.77877 2.90440 3.96766 3.09330 3.92946 2.81769 1.93554 2.79861 2.97212 3.22924 5.34353 4.06792 107 k - - -

2.68618 4.42225 2.77519 2.73123 3.46354 2.40513 3.72494 3.29354 2.67741 2.69355 4.24690 2.90347 2.73739 3.18146 2.89801 2.37887 2.77519 2.98518 4.58477 3.61503

0.02318 4.17189 4.89423 0.61958 0.77255 0.48576 0.95510

99 2.02966 3.46567 3.92077 3.35031 3.03871 3.64650 4.01458 2.16251 3.25728 2.35090 3.28285 3.60853 4.05033 3.50603 3.49459 2.38891 2.84310 2.17521 4.79809 3.59658 108 a - - -

2.68618 4.42225 2.77519 2.73123 3.46354 2.40513 3.72494 3.29354 2.67741 2.69355 4.24690 2.90347 2.73739 3.18146 2.89801 2.37887 2.77519 2.98518 4.58477 3.61503

0.01566 4.16437 * 0.61958 0.77255 0.00000 *

//

GCNA ZINC FINGER HMM

HMMER3/f [3.1b1 | May 2013]

NAME GCNAsprtsCxxConlyUPDATE

LENG 30

ALPH amino

RF no

MM no

CONS yes

CS no

MAP yes

DATE Mon Mar 9 15:16:55 2015

NSEQ 14

EFFN 6.634277

CKSUM 3862321358

STATS LOCAL MSV -7.2577 0.71988

STATS LOCAL VITERBI -7.3586 0.71988

STATS LOCAL FORWARD -3.7740 0.71988

HMM A C D E F G H I K L M N P Q R S T V W Y

m->m m->i m->d i->m i->i d->m d->d

COMPO 3.04710 1.98070 2.96527 3.04761 3.96510 2.53811 3.52342 3.24077 2.44486 3.14208 4.45888 3.43478 4.20287 3.34080 2.51945 2.32385 3.02787 3.08080 4.37118 3.23349

2.68618 4.42225 2.77519 2.73123 3.46354 2.40513 3.72494 3.29354 2.67741 2.69355 4.24690 2.90347 2.73739 3.18146 2.89801 2.37887 2.77519 2.98518 4.58477 3.61503

0.00554 5.59354 6.31589 0.61958 0.77255 0.00000 *

1 5.88227 6.65649 6.50274 6.55444 3.09269 6.13914 4.70834 5.42880 6.29158 4.47096 5.90683 5.66305 6.39905 5.86947 6.02342 5.62287 6.06494 5.40274 1.26005 0.49170 1 Y - - -

2.68618 4.42225 2.77519 2.73123 3.46354 2.40513 3.72494 3.29354 2.67741 2.69355 4.24690 2.90347 2.73739 3.18146 2.89801 2.37887 2.77519 2.98518 4.58477 3.61503

0.00554 5.59354 6.31589 0.61958 0.77255 0.48576 0.95510

2 2.68733 5.62173 3.44530 2.19498 4.94320 3.95458 4.14295 3.10566 1.64221 2.90043 4.66890 3.43205 4.34722 2.37171 2.02655 2.66326 3.40264 2.89816 6.06820 4.67347 2 k - - -

2.68618 4.42225 2.77519 2.73123 3.46354 2.40513 3.72494 3.29354 2.67741 2.69355 4.24690 2.90347 2.73739 3.18146 2.89801 2.37887 2.77519 2.98518 4.58477 3.61503

0.00554 5.59354 6.31589 0.61958 0.77255 0.48576 0.95510

3 5.54397 0.02966 6.72051 6.78783 6.97495 5.56799 7.25194 6.89991 6.83881 6.39422 7.60193 6.72839 6.28671 7.11142 6.65323 5.81607 6.08470 6.41703 7.67272 7.19965 3 C - - -

2.68618 4.42225 2.77519 2.73123 3.46354 2.40513 3.72494 3.29354 2.67741 2.69355 4.24690 2.90347 2.73739 3.18146 2.89801 2.37887 2.77519 2.98518 4.58477 3.61503

0.50435 5.59354 0.93552 0.61958 0.77255 0.48576 0.95510

4 3.00877 5.18382 3.33098 2.18604 4.43183 3.80498 4.03380 2.61772 2.81993 3.44021 4.27028 3.34677 4.21476 2.16911 3.26105 3.04613 1.30866 3.50159 5.69949 4.37197 4 t - - -

2.68672 4.42279 2.77436 2.73046 3.46408 2.40567 3.72549 3.29408 2.67724 2.69409 4.24744 2.90167 2.73794 3.17994 2.89777 2.37869 2.77574 2.98572 4.58531 3.61557

1.74638 0.19525 5.82065 0.38992 1.13044 0.13756 2.05166

5 2.64245 5.62669 2.28761 2.08646 4.94943 2.47524 4.14331 4.41988 2.88840 2.08447 4.67405 2.40419 4.34601 3.24763 3.37745 2.66221 2.41905 3.11547 6.07367 4.67647 8 l - - -

2.68618 4.42225 2.77519 2.73123 3.46354 2.40513 3.72494 3.29354 2.67741 2.69355 4.24690 2.90347 2.73739 3.18146 2.89801 2.37887 2.77519 2.98518 4.58477 3.61503

0.00554 5.59354 6.31589 0.61958 0.77255 0.48576 0.95510

6 3.17557 5.64984 3.43771 2.13687 4.98199 1.55599 4.13976 4.45695 2.39049 3.94840 4.69454 3.42653 4.34631 2.73543 2.81327 1.91201 3.40626 3.12634 3.35754 4.68917 9 g - - -

2.68618 4.42225 2.77519 2.73123 3.46354 2.40513 3.72494 3.29354 2.67741 2.69355 4.24690 2.90347 2.73739 3.18146 2.89801 2.37887 2.77519 2.98518 4.58477 3.61503

0.00554 5.59354 6.31589 0.61958 0.77255 0.48576 0.95510

7 5.54397 0.02966 6.72051 6.78783 6.97495 5.56799 7.25194 6.89991 6.83881 6.39422 7.60193 6.72839 6.28671 7.11142 6.65323 5.81607 6.08470 6.41703 7.67272 7.19965 10 C - - -

2.68618 4.42225 2.77519 2.73123 3.46354 2.40513 3.72494 3.29354 2.67741 2.69355 4.24690 2.90347 2.73739 3.18146 2.89801 2.37887 2.77519 2.98518 4.58477 3.61503

0.00554 5.59354 6.31589 0.61958 0.77255 0.48576 0.95510

8 3.55354 6.12923 1.53975 2.39773 5.45575 1.56969 4.44782 4.96399 1.76741 4.42590 5.19118 2.63015 4.60577 2.67785 3.81610 3.47758 3.80336 4.51295 6.55475 5.09746 11 d - - -

2.68618 4.42225 2.77519 2.73123 3.46354 2.40513 3.72494 3.29354 2.67741 2.69355 4.24690 2.90347 2.73739 3.18146 2.89801 2.37887 2.77519 2.98518 4.58477 3.61503

0.00554 5.59354 6.31589 0.61958 0.77255 0.48576 0.95510

9 2.68890 5.63072 3.44733 2.89006 4.95523 3.95703 3.09760 4.42492 1.72469 3.92591 4.67731 2.85494 4.34951 2.32082 2.30643 3.14858 2.76097 1.97525 6.07469 3.35105 12 k - - -

2.68618 4.42225 2.77519 2.73123 3.46354 2.40513 3.72494 3.29354 2.67741 2.69355 4.24690 2.90347 2.73739 3.18146 2.89801 2.37887 2.77519 2.98518 4.58477 3.61503

0.00554 5.59354 6.31589 0.61958 0.77255 0.48576 0.95510

10 2.33251 5.30012 3.64277 1.52384 4.51607 4.03677 4.24722 2.92863 3.07290 2.73565 4.37821 3.59201 4.42541 3.41797 2.44482 2.39899 2.17954 2.86656 5.81998 4.50444 13 e - - -

2.68618 4.42225 2.77519 2.73123 3.46354 2.40513 3.72494 3.29354 2.67741 2.69355 4.24690 2.90347 2.73739 3.18146 2.89801 2.37887 2.77519 2.98518 4.58477 3.61503

0.00554 5.59354 6.31589 0.61958 0.77255 0.48576 0.95510

11 3.40564 4.77183 5.32044 4.72748 1.86473 4.56702 4.79082 1.67645 4.52022 2.92265 3.88393 4.69928 4.92536 4.64145 4.53303 2.45039 3.63829 2.09103 5.22029 1.51223 14 y - - -

2.68618 4.42225 2.77519 2.73123 3.46354 2.40513 3.72494 3.29354 2.67741 2.69355 4.24690 2.90347 2.73739 3.18146 2.89801 2.37887 2.77519 2.98518 4.58477 3.61503

0.00554 5.59354 6.31589 0.61958 0.77255 0.48576 0.95510

12 4.20055 6.33279 3.96538 3.80221 5.93095 0.42925 2.39768 5.54986 2.43119 4.96778 5.86198 4.26073 5.17964 4.19261 3.72514 4.19233 4.50788 5.11055 6.89168 5.62482 15 G - - -

2.68618 4.42225 2.77519 2.73123 3.46354 2.40513 3.72494 3.29354 2.67741 2.69355 4.24690 2.90347 2.73739 3.18146 2.89801 2.37887 2.77519 2.98518 4.58477 3.61503

0.00554 5.59354 6.31589 0.61958 0.77255 0.48576 0.95510

13 2.16990 2.95222 5.47507 4.92214 5.79676 4.30605 5.48146 5.15437 3.93723 4.80354 5.66064 4.80862 5.05534 4.73119 0.37407 3.79465 4.09393 4.58208 6.94776 5.86674 16 R - - -

2.68618 4.42225 2.77519 2.73123 3.46354 2.40513 3.72494 3.29354 2.67741 2.69355 4.24690 2.90347 2.73739 3.18146 2.89801 2.37887 2.77519 2.98518 4.58477 3.61503

0.00554 5.59354 6.31589 0.61958 0.77255 0.48576 0.95510

14 3.41913 5.77620 3.52300 3.08556 4.76815 4.12750 1.02929 4.53566 3.10771 4.05140 4.85406 2.27729 4.55634 2.61067 3.55819 2.38261 3.65453 4.16004 6.04472 2.58697 17 h - - -

2.68618 4.42225 2.77519 2.73123 3.46354 2.40513 3.72494 3.29354 2.67741 2.69355 4.24690 2.90347 2.73739 3.18146 2.89801 2.37887 2.77519 2.98518 4.58477 3.61503

0.00554 5.59354 6.31589 0.61958 0.77255 0.48576 0.95510

15 3.37434 5.24220 5.17974 4.85467 5.33403 4.13509 5.63551 4.69650 4.75775 4.41570 2.23438 4.73185 4.91578 5.01517 4.95305 0.60276 1.66626 4.20857 6.76339 5.60284 18 S - - -

2.68618 4.42225 2.77519 2.73123 3.46354 2.40513 3.72494 3.29354 2.67741 2.69355 4.24690 2.90347 2.73739 3.18146 2.89801 2.37887 2.77519 2.98518 4.58477 3.61503

0.00554 5.59354 6.31589 0.61958 0.77255 0.48576 0.95510

16 4.55896 6.53564 5.31124 4.26650 6.27908 5.02000 4.70879 5.46602 0.42096 4.73315 5.64425 4.53081 2.52486 3.83254 2.00380 4.52113 4.62386 5.16840 6.63027 5.62355 19 K - - -

2.68618 4.42225 2.77519 2.73123 3.46354 2.40513 3.72494 3.29354 2.67741 2.69355 4.24690 2.90347 2.73739 3.18146 2.89801 2.37887 2.77519 2.98518 4.58477 3.61503

0.09938 5.59354 2.39818 0.61958 0.77255 0.48576 0.95510

17 3.37720 2.07365 5.80037 5.78508 6.02763 4.08457 6.25259 5.57065 5.63698 5.29676 6.06922 5.02003 4.96299 5.73684 5.59024 0.29152 3.97343 4.68071 7.36952 6.33237 20 S - - -

2.68618 4.42225 2.77519 2.73123 3.46354 2.40513 3.72494 3.29354 2.67741 2.69355 4.24690 2.90347 2.73739 3.18146 2.89801 2.37887 2.77519 2.98518 4.58477 3.61503

0.00609 5.50025 6.22260 0.61958 0.77255 0.86058 0.54977

18 3.91009 5.20069 5.86092 5.28336 4.11485 5.23400 5.62592 0.95531 5.06711 1.27015 3.95121 5.37123 5.49354 5.22112 2.46223 4.58556 4.14381 2.16590 5.99655 4.87358 21 i - - -

2.68618 4.42225 2.77519 2.73123 3.46354 2.40513 3.72494 3.29354 2.67741 2.69355 4.24690 2.90347 2.73739 3.18146 2.89801 2.37887 2.77519 2.98518 4.58477 3.61503

0.00609 5.50025 6.22260 0.61958 0.77255 0.30720 1.32992

19 4.30934 7.10468 0.58975 3.18194 6.36964 4.34764 5.00855 5.97826 2.31863 5.38982 6.30960 1.61835 5.04677 4.19397 4.84707 4.11733 4.62941 5.48294 7.51972 5.88961 22 D - - -

2.68618 4.42225 2.77519 2.73123 3.46354 2.40513 3.72494 3.29354 2.67741 2.69355 4.24690 2.90347 2.73739 3.18146 2.89801 2.37887 2.77519 2.98518 4.58477 3.61503

0.00554 5.59354 6.31589 0.61958 0.77255 0.48576 0.95510

20 3.23377 4.84877 2.67342 3.73985 3.97686 4.26031 4.54030 2.62092 3.67503 2.56905 3.96200 4.08595 2.47263 3.95211 3.97550 2.71743 1.66168 1.56544 5.45363 4.23811 23 v - - -

2.68618 4.42225 2.77519 2.73123 3.46354 2.40513 3.72494 3.29354 2.67741 2.69355 4.24690 2.90347 2.73739 3.18146 2.89801 2.37887 2.77519 2.98518 4.58477 3.61503

0.00554 5.59354 6.31589 0.61958 0.77255 0.48576 0.95510

21 2.59728 5.68889 2.77500 1.67167 5.03458 3.94312 4.13371 4.52165 2.10693 3.99443 4.72974 2.71542 4.33915 2.59507 3.36385 1.99510 2.14032 4.07993 6.12089 4.70845 24 e - - -

2.68618 4.42225 2.77519 2.73123 3.46354 2.40513 3.72494 3.29354 2.67741 2.69355 4.24690 2.90347 2.73739 3.18146 2.89801 2.37887 2.77519 2.98518 4.58477 3.61503

0.00554 5.59354 6.31589 0.61958 0.77255 0.48576 0.95510

22 3.38379 5.75434 2.45536 3.11009 5.09756 4.14144 4.26634 4.52711 1.45155 2.59608 4.80815 3.62645 4.52444 3.37941 1.30007 3.36405 3.60048 2.94489 6.15970 4.82420 25 r - - -

2.68618 4.42225 2.77519 2.73123 3.46354 2.40513 3.72494 3.29354 2.67741 2.69355 4.24690 2.90347 2.73739 3.18146 2.89801 2.37887 2.77519 2.98518 4.58477 3.61503

0.00554 5.59354 6.31589 0.61958 0.77255 0.48576 0.95510

23 3.38758 5.33758 3.97232 3.39029 2.23894 4.25022 2.07757 3.90689 2.10224 1.71939 4.41545 3.84866 4.62734 1.77826 3.50730 3.49142 3.60900 3.64998 5.72708 4.38419 26 l - - -

2.68618 4.42225 2.77519 2.73123 3.46354 2.40513 3.72494 3.29354 2.67741 2.69355 4.24690 2.90347 2.73739 3.18146 2.89801 2.37887 2.77519 2.98518 4.58477 3.61503

0.00554 5.59354 6.31589 0.61958 0.77255 0.48576 0.95510

24 2.14206 3.22023 5.10738 4.50035 3.74328 4.41269 4.74302 2.20556 4.30934 2.83576 3.76597 4.53841 4.77771 4.46272 2.59876 3.71977 2.68454 1.20770 5.27143 2.60355 27 v - - -

2.68618 4.42225 2.77519 2.73123 3.46354 2.40513 3.72494 3.29354 2.67741 2.69355 4.24690 2.90347 2.73739 3.18146 2.89801 2.37887 2.77519 2.98518 4.58477 3.61503

0.00554 5.59354 6.31589 0.61958 0.77255 0.48576 0.95510

25 5.54397 0.02966 6.72051 6.78783 6.97495 5.56799 7.25194 6.89991 6.83881 6.39422 7.60193 6.72839 6.28671 7.11142 6.65323 5.81607 6.08470 6.41703 7.67272 7.19965 28 C - - -

2.68618 4.42225 2.77519 2.73123 3.46354 2.40513 3.72494 3.29354 2.67741 2.69355 4.24690 2.90347 2.73739 3.18146 2.89801 2.37887 2.77519 2.98518 4.58477 3.61503

0.00554 5.59354 6.31589 0.61958 0.77255 0.48576 0.95510

26 1.56464 5.29431 4.08435 3.55521 4.85932 1.38817 4.66085 2.87325 3.50218 3.90686 4.73603 3.95963 4.62164 3.85781 2.00674 2.14783 3.61223 3.89258 6.17263 4.89517 29 g - - -

2.68618 4.42225 2.77519 2.73123 3.46354 2.40513 3.72494 3.29354 2.67741 2.69355 4.24690 2.90347 2.73739 3.18146 2.89801 2.37887 2.77519 2.98518 4.58477 3.61503

0.00554 5.59354 6.31589 0.61958 0.77255 0.48576 0.95510

27 2.59256 5.40824 2.36053 3.00574 3.32129 4.00484 4.20646 2.63019 2.02734 2.44370 4.47657 3.52860 4.39499 3.35044 1.68480 2.60939 3.41093 3.74443 5.90501 4.56358 30 r - - -

2.68618 4.42225 2.77519 2.73123 3.46354 2.40513 3.72494 3.29354 2.67741 2.69355 4.24690 2.90347 2.73739 3.18146 2.89801 2.37887 2.77519 2.98518 4.58477 3.61503

0.00554 5.59354 6.31589 0.61958 0.77255 0.48576 0.95510

28 5.54397 0.02966 6.72051 6.78783 6.97495 5.56799 7.25194 6.89991 6.83881 6.39422 7.60193 6.72839 6.28671 7.11142 6.65323 5.81607 6.08470 6.41703 7.67272 7.19965 31 C - - -

2.68618 4.42225 2.77519 2.73123 3.46354 2.40513 3.72494 3.29354 2.67741 2.69355 4.24690 2.90347 2.73739 3.18146 2.89801 2.37887 2.77519 2.98518 4.58477 3.61503

0.00554 5.59354 6.31589 0.61958 0.77255 0.48576 0.95510

29 3.18433 5.66957 2.72646 2.88401 3.12955 2.04883 3.09429 4.48549 1.74334 3.96975 4.71326 3.42923 4.35145 3.24254 1.67286 3.15068 2.84237 4.05688 6.10423 4.70256 32 r - - -

2.68618 4.42225 2.77519 2.73123 3.46354 2.40513 3.72494 3.29354 2.67741 2.69355 4.24690 2.90347 2.73739 3.18146 2.89801 2.37887 2.77519 2.98518 4.58477 3.61503

0.00554 5.59354 6.31589 0.61958 0.77255 0.48576 0.95510

30 3.52243 5.44224 5.30632 5.34857 6.30033 0.67426 6.26484 5.89006 5.61283 5.55214 6.29472 4.99617 5.07243 5.68107 5.67337 0.95320 4.11686 4.90418 7.57740 6.56784 33 G - - -

2.68618 4.42225 2.77519 2.73123 3.46354 2.40513 3.72494 3.29354 2.67741 2.69355 4.24690 2.90347 2.73739 3.18146 2.89801 2.37887 2.77519 2.98518 4.58477 3.61503

0.00374 5.59174 * 0.61958 0.77255 0.00000 *

//

GCNA HMG HMM

HMMER3/f [3.1b1 | May 2013]

NAME GCNAhmgUPDATE

LENG 49

ALPH amino

RF no

MM no

CONS yes

CS no

MAP yes

DATE Mon Mar 9 15:18:29 2015

NSEQ 14

EFFN 7.755371

CKSUM 3768781407

STATS LOCAL MSV -7.9616 0.71921

STATS LOCAL VITERBI -8.1807 0.71921

STATS LOCAL FORWARD -4.4007 0.71921

HMM A C D E F G H I K L M N P Q R S T V W Y

m->m m->i m->d i->m i->i d->m d->d

COMPO 2.44686 5.69478 3.09909 2.48541 2.89180 3.28406 3.36910 3.41520 2.18260 2.60564 3.55464 2.96150 3.30001 2.88712 2.78625 2.80146 2.96303 3.00962 4.68157 3.53079

2.68618 4.42225 2.77519 2.73123 3.46354 2.40513 3.72494 3.29354 2.67741 2.69355 4.24690 2.90347 2.73739 3.18146 2.89801 2.37887 2.77519 2.98518 4.58477 3.61503

0.00482 5.73338 6.45573 0.61958 0.77255 0.00000 *

1 2.13709 5.70934 3.52000 2.37055 5.03338 4.03269 4.22187 2.58329 1.44781 4.00537 4.75578 2.64360 2.92417 2.57259 3.45215 3.22379 3.48235 2.72976 6.15464 4.75733 1 k - - -

2.68618 4.42225 2.77519 2.73123 3.46354 2.40513 3.72494 3.29354 2.67741 2.69355 4.24690 2.90347 2.73739 3.18146 2.89801 2.37887 2.77519 2.98518 4.58477 3.61503

0.00482 5.73338 6.45573 0.61958 0.77255 0.48576 0.95510

2 1.89482 5.62616 3.56476 2.40749 4.91809 4.05259 4.24574 2.98522 1.63150 3.90828 4.68171 3.54503 3.20046 3.36298 2.77818 2.43416 3.48692 2.14280 6.09218 4.71686 2 k - - -

2.68618 4.42225 2.77519 2.73123 3.46354 2.40513 3.72494 3.29354 2.67741 2.69355 4.24690 2.90347 2.73739 3.18146 2.89801 2.37887 2.77519 2.98518 4.58477 3.61503

0.00482 5.73338 6.45573 0.61958 0.77255 0.48576 0.95510

3 3.36734 5.85211 3.60897 1.84192 5.21539 2.80400 4.28239 4.68649 1.94233 4.14949 4.89726 3.59025 2.53286 3.38189 1.34662 3.32896 2.80123 4.25454 6.26148 4.87750 3 r - - -

2.68618 4.42225 2.77519 2.73123 3.46354 2.40513 3.72494 3.29354 2.67741 2.69355 4.24690 2.90347 2.73739 3.18146 2.89801 2.37887 2.77519 2.98518 4.58477 3.61503

0.00482 5.73338 6.45573 0.61958 0.77255 0.48576 0.95510

4 1.51153 5.79188 3.49290 2.36376 5.12935 4.05395 2.03469 4.60990 3.00294 4.09465 4.83985 2.78508 4.45937 2.14800 3.49399 3.26420 2.04170 4.17958 6.23103 4.82333 4 a - - -

2.68618 4.42225 2.77519 2.73123 3.46354 2.40513 3.72494 3.29354 2.67741 2.69355 4.24690 2.90347 2.73739 3.18146 2.89801 2.37887 2.77519 2.98518 4.58477 3.61503

0.00482 5.73338 6.45573 0.61958 0.77255 0.48576 0.95510

5 2.50111 5.18723 4.23971 2.40608 4.42316 4.34304 4.74626 3.63550 3.75309 2.67618 4.37118 4.15497 0.86638 4.06041 4.10845 3.64524 3.69988 2.20952 5.87769 4.64016 5 p - - -

2.68618 4.42225 2.77519 2.73123 3.46354 2.40513 3.72494 3.29354 2.67741 2.69355 4.24690 2.90347 2.73739 3.18146 2.89801 2.37887 2.77519 2.98518 4.58477 3.61503

0.00482 5.73338 6.45573 0.61958 0.77255 0.48576 0.95510

6 3.30096 5.80162 3.52963 2.29907 5.15171 4.06195 4.24540 4.63247 2.35631 4.10409 4.84518 1.59651 4.45846 3.34384 2.31696 2.34181 1.63481 4.19587 6.22744 4.82519 6 n - - -

2.68618 4.42225 2.77519 2.73123 3.46354 2.40513 3.72494 3.29354 2.67741 2.69355 4.24690 2.90347 2.73739 3.18146 2.89801 2.37887 2.77519 2.98518 4.58477 3.61503

0.00482 5.73338 6.45573 0.61958 0.77255 0.48576 0.95510

7 1.19327 5.49496 4.58883 4.13922 5.71798 1.65748 5.26263 5.19058 4.11059 4.78093 5.54168 4.39975 1.68238 4.44501 2.38787 2.33855 3.90809 4.55302 6.94449 5.68836 7 a - - -

2.68618 4.42225 2.77519 2.73123 3.46354 2.40513 3.72494 3.29354 2.67741 2.69355 4.24690 2.90347 2.73739 3.18146 2.89801 2.37887 2.77519 2.98518 4.58477 3.61503

0.00482 5.73338 6.45573 0.61958 0.77255 0.48576 0.95510

8 5.99905 6.77156 6.63739 6.69902 0.18751 6.28820 4.81149 5.49542 6.46124 4.52995 5.97390 5.77577 6.53117 5.98702 6.17206 5.73568 6.17941 5.48584 4.87279 2.21122 8 F - - -

2.68618 4.42225 2.77519 2.73123 3.46354 2.40513 3.72494 3.29354 2.67741 2.69355 4.24690 2.90347 2.73739 3.18146 2.89801 2.37887 2.77519 2.98518 4.58477 3.61503

0.00482 5.73338 6.45573 0.61958 0.77255 0.48576 0.95510

9 1.05682 5.89166 2.38027 3.01932 5.24492 4.09725 4.33912 4.73154 2.32272 4.20943 4.95744 3.57134 4.52713 2.13228 3.59348 2.20354 3.62795 4.29255 6.34135 4.92519 9 a - - -

2.68618 4.42225 2.77519 2.73123 3.46354 2.40513 3.72494 3.29354 2.67741 2.69355 4.24690 2.90347 2.73739 3.18146 2.89801 2.37887 2.77519 2.98518 4.58477 3.61503

0.00482 5.73338 6.45573 0.61958 0.77255 0.48576 0.95510

10 3.24998 5.74752 3.50398 2.29455 5.08793 4.02529 4.21187 3.03942 1.87947 2.45158 4.78959 2.63912 4.41848 2.54778 2.07674 2.11846 2.82146 4.13569 6.18252 4.77558 10 k - - -

2.68618 4.42225 2.77519 2.73123 3.46354 2.40513 3.72494 3.29354 2.67741 2.69355 4.24690 2.90347 2.73739 3.18146 2.89801 2.37887 2.77519 2.98518 4.58477 3.61503

0.00482 5.73338 6.45573 0.61958 0.77255 0.48576 0.95510

11 6.01639 6.77490 6.64328 6.70953 0.46762 6.29978 4.79900 5.51682 6.46805 4.55009 5.99336 5.76953 6.53587 5.98301 6.17340 5.73718 6.19152 5.50119 4.85979 1.16341 11 F - - -

2.68618 4.42225 2.77519 2.73123 3.46354 2.40513 3.72494 3.29354 2.67741 2.69355 4.24690 2.90347 2.73739 3.18146 2.89801 2.37887 2.77519 2.98518 4.58477 3.61503

0.00482 5.73338 6.45573 0.61958 0.77255 0.48576 0.95510

12 3.83005 5.16472 5.79898 5.21893 4.16788 5.07297 5.48075 3.01812 5.03415 1.47392 2.64985 5.24836 5.39888 5.15690 5.06265 2.60072 4.07176 0.77941 5.90682 4.75658 12 v - - -

2.68618 4.42225 2.77519 2.73123 3.46354 2.40513 3.72494 3.29354 2.67741 2.69355 4.24690 2.90347 2.73739 3.18146 2.89801 2.37887 2.77519 2.98518 4.58477 3.61503

0.00482 5.73338 6.45573 0.61958 0.77255 0.48576 0.95510

13 4.45114 6.56603 5.00109 2.47948 6.21143 4.98390 4.73644 5.45349 0.67776 4.74922 5.62807 4.46107 5.29366 2.19803 1.64392 4.40368 4.54709 5.13011 6.67942 5.60590 13 K - - -

2.68618 4.42225 2.77519 2.73123 3.46354 2.40513 3.72494 3.29354 2.67741 2.69355 4.24690 2.90347 2.73739 3.18146 2.89801 2.37887 2.77519 2.98518 4.58477 3.61503

0.00482 5.73338 6.45573 0.61958 0.77255 0.48576 0.95510

14 4.10761 6.76865 1.20560 0.86230 6.05938 4.37255 4.91390 5.60523 3.94201 2.84790 5.89918 3.81999 4.99537 4.07217 4.56526 2.57567 4.39528 5.14440 7.20984 5.66845 14 e - - -

2.68618 4.42225 2.77519 2.73123 3.46354 2.40513 3.72494 3.29354 2.67741 2.69355 4.24690 2.90347 2.73739 3.18146 2.89801 2.37887 2.77519 2.98518 4.58477 3.61503

0.00482 5.73338 6.45573 0.61958 0.77255 0.48576 0.95510

15 4.17834 6.47663 4.10659 3.68764 5.91712 4.68128 2.59254 5.34185 3.07163 4.71296 5.56247 0.55099 5.10617 2.44710 2.52082 4.11591 4.35717 4.97195 6.69804 5.46358 15 N - - -

2.68618 4.42225 2.77519 2.73123 3.46354 2.40513 3.72494 3.29354 2.67741 2.69355 4.24690 2.90347 2.73739 3.18146 2.89801 2.37887 2.77519 2.98518 4.58477 3.61503

0.00482 5.73338 6.45573 0.61958 0.77255 0.48576 0.95510

16 5.07519 6.20877 6.32148 6.09509 0.73756 5.83744 4.80444 4.76436 5.87571 4.12272 2.68192 5.54867 6.13224 5.66829 5.74405 2.76999 5.29281 4.66064 4.90742 1.18722 16 f - - -

2.68618 4.42225 2.77519 2.73123 3.46354 2.40513 3.72494 3.29354 2.67741 2.69355 4.24690 2.90347 2.73739 3.18146 2.89801 2.37887 2.77519 2.98518 4.58477 3.61503

0.00482 5.73338 6.45573 0.61958 0.77255 0.48576 0.95510

17 2.05711 5.75543 3.51078 2.95404 5.09816 1.97476 4.21591 4.57980 2.03481 4.05832 4.79765 2.44674 4.42424 2.54863 2.33392 2.50338 3.48782 4.14502 2.98372 4.78271 17 g - - -

2.68618 4.42225 2.77519 2.73123 3.46354 2.40513 3.72494 3.29354 2.67741 2.69355 4.24690 2.90347 2.73739 3.18146 2.89801 2.37887 2.77519 2.98518 4.58477 3.61503

0.00482 5.73338 6.45573 0.61958 0.77255 0.48576 0.95510

18 1.66548 5.68645 3.56209 2.23773 4.99840 4.05860 4.24340 2.93121 1.77097 2.98632 4.73740 3.54207 4.45013 3.35329 1.89910 2.69048 3.50229 4.05933 6.13699 4.75441 18 a - - -

2.68618 4.42225 2.77519 2.73123 3.46354 2.40513 3.72494 3.29354 2.67741 2.69355 4.24690 2.90347 2.73739 3.18146 2.89801 2.37887 2.77519 2.98518 4.58477 3.61503

0.00482 5.73338 6.45573 0.61958 0.77255 0.48576 0.95510

19 2.51624 4.74653 5.12264 4.52009 3.83949 4.48870 2.90016 3.17727 4.34214 1.72281 3.86425 4.59016 4.85515 4.50846 4.41465 3.79251 1.54781 1.60447 5.36464 2.66324 19 t - - -

2.68618 4.42225 2.77519 2.73123 3.46354 2.40513 3.72494 3.29354 2.67741 2.69355 4.24690 2.90347 2.73739 3.18146 2.89801 2.37887 2.77519 2.98518 4.58477 3.61503

0.00482 5.73338 6.45573 0.61958 0.77255 0.48576 0.95510

20 2.48345 5.90290 4.06764 3.43721 5.27816 4.39882 4.47520 4.67266 0.70422 4.17757 2.97106 3.91527 4.77530 3.60058 2.48952 3.65330 2.53639 4.31983 6.30605 5.03231 20 k - - -

2.68618 4.42225 2.77519 2.73123 3.46354 2.40513 3.72494 3.29354 2.67741 2.69355 4.24690 2.90347 2.73739 3.18146 2.89801 2.37887 2.77519 2.98518 4.58477 3.61503

0.08962 5.73338 2.49517 0.61958 0.77255 0.48576 0.95510

21 1.99635 5.73577 2.60872 2.94768 5.08302 4.01941 4.19277 4.55782 1.96293 2.54408 4.77911 3.48962 4.41110 2.01007 1.69618 3.21599 3.47918 4.12829 6.15998 4.76569 21 r - - -

2.68618 4.42225 2.77519 2.73123 3.46354 2.40513 3.72494 3.29354 2.67741 2.69355 4.24690 2.90347 2.73739 3.18146 2.89801 2.37887 2.77519 2.98518 4.58477 3.61503

0.00524 5.64901 6.37136 0.61958 0.77255 0.87445 0.53973

22 1.85021 5.72201 2.57552 1.70611 5.06704 2.66810 4.16777 4.55380 2.17041 2.85644 4.76308 2.58519 4.37291 2.55981 3.39861 3.17003 3.44051 4.11270 6.15428 4.74198 22 e - - -

2.68618 4.42225 2.77519 2.73123 3.46354 2.40513 3.72494 3.29354 2.67741 2.69355 4.24690 2.90347 2.73739 3.18146 2.89801 2.37887 2.77519 2.98518 4.58477 3.61503

0.00524 5.64901 6.37136 0.61958 0.77255 0.30226 1.34378

23 2.43780 5.62152 3.56550 2.55038 4.91156 4.05266 2.21825 2.99394 3.00595 1.97964 4.67720 1.86834 4.44459 2.71737 2.76378 3.24734 2.84442 3.98225 6.08843 4.71389 23 n - - -

2.68618 4.42225 2.77519 2.73123 3.46354 2.40513 3.72494 3.29354 2.67741 2.69355 4.24690 2.90347 2.73739 3.18146 2.89801 2.37887 2.77519 2.98518 4.58477 3.61503

0.07741 5.73338 2.64154 0.61958 0.77255 0.48576 0.95510

24 3.57988 6.05493 3.39447 2.05088 5.45380 2.68676 4.52766 4.94355 3.34334 4.42996 5.20448 3.66377 0.96426 2.04244 3.84703 3.52932 2.83133 4.50059 6.57133 5.14394 24 p - - -

2.68545 4.42238 2.77532 2.73136 3.46366 2.40525 3.72507 3.29366 2.67753 2.69367 4.24702 2.90359 2.73646 3.18159 2.89813 2.37899 2.77532 2.98531 4.58489 3.61516

0.17885 1.81966 6.38350 0.08944 2.45861 0.82871 0.57378

25 2.48614 5.47962 3.61682 3.06071 4.76110 1.44503 2.90484 4.18777 3.04545 2.69411 4.57173 3.58170 4.44656 3.40454 2.14709 2.30678 2.57106 3.84266 5.99472 4.64936 26 g - - -

2.68618 4.42225 2.77519 2.73123 3.46354 2.40513 3.72494 3.29354 2.67741 2.69355 4.24690 2.90347 2.73739 3.18146 2.89801 2.37887 2.77519 2.98518 4.58477 3.61503

0.00518 5.66116 6.38350 0.61958 0.77255 0.31892 1.29805

26 3.80857 5.16013 5.69556 5.08957 3.97814 5.00699 5.32928 1.78223 4.89526 0.85358 2.60883 5.14460 5.30664 3.13989 4.91583 4.32784 2.04313 3.22549 5.73930 4.61861 27 l - - -

2.68618 4.42225 2.77519 2.73123 3.46354 2.40513 3.72494 3.29354 2.67741 2.69355 4.24690 2.90347 2.73739 3.18146 2.89801 2.37887 2.77519 2.98518 4.58477 3.61503

0.00482 5.73338 6.45573 0.61958 0.77255 0.48576 0.95510

27 3.31464 5.80657 3.49766 2.52396 5.15621 4.06155 4.27439 4.63839 2.30733 4.11999 4.86441 2.69073 2.26602 3.37636 3.50750 1.73104 1.41619 4.20364 6.25345 4.84498 28 t - - -

2.68618 4.42225 2.77519 2.73123 3.46354 2.40513 3.72494 3.29354 2.67741 2.69355 4.24690 2.90347 2.73739 3.18146 2.89801 2.37887 2.77519 2.98518 4.58477 3.61503

0.00482 5.73338 6.45573 0.61958 0.77255 0.48576 0.95510

28 2.39717 5.50980 4.70994 4.45214 5.70077 4.25911 0.52178 5.17306 4.47609 4.82329 5.63669 4.61097 2.40008 4.80632 4.77353 3.72574 2.41732 4.57870 7.01805 5.80108 29 H - - -

2.68618 4.42225 2.77519 2.73123 3.46354 2.40513 3.72494 3.29354 2.67741 2.69355 4.24690 2.90347 2.73739 3.18146 2.89801 2.37887 2.77519 2.98518 4.58477 3.61503

0.00482 5.73338 6.45573 0.61958 0.77255 0.48576 0.95510

29 1.76861 5.78225 2.43657 2.96755 5.13470 2.15251 4.23969 4.61892 1.53534 4.09431 4.83327 3.51466 4.44318 3.33816 2.52090 2.42493 3.51347 4.17953 6.22212 4.81361 30 k - - -

2.68618 4.42225 2.77519 2.73123 3.46354 2.40513 3.72494 3.29354 2.67741 2.69355 4.24690 2.90347 2.73739 3.18146 2.89801 2.37887 2.77519 2.98518 4.58477 3.61503

0.00482 5.73338 6.45573 0.61958 0.77255 0.48576 0.95510

30 2.90584 7.06466 1.14203 0.83017 6.32640 4.42322 5.03906 5.91083 4.16302 5.33886 6.21418 3.85961 5.09284 2.59131 4.85015 4.12459 4.60077 5.42485 7.48097 5.87466 31 e - - -

2.68618 4.42225 2.77519 2.73123 3.46354 2.40513 3.72494 3.29354 2.67741 2.69355 4.24690 2.90347 2.73739 3.18146 2.89801 2.37887 2.77519 2.98518 4.58477 3.61503

0.00482 5.73338 6.45573 0.61958 0.77255 0.48576 0.95510

31 4.55955 5.70500 6.73260 6.29129 4.83475 6.31846 7.14921 1.92674 6.28299 1.35381 4.51810 6.49741 6.41138 6.43389 6.41890 5.80475 4.81299 0.62487 7.17323 6.05407 32 V - - -

2.68618 4.42225 2.77519 2.73123 3.46354 2.40513 3.72494 3.29354 2.67741 2.69355 4.24690 2.90347 2.73739 3.18146 2.89801 2.37887 2.77519 2.98518 4.58477 3.61503

0.00482 5.73338 6.45573 0.61958 0.77255 0.48576 0.95510

32 4.73344 6.42505 5.48443 4.95905 5.49667 5.23606 5.51714 4.90685 2.19634 4.18343 0.26431 5.19155 5.73815 4.75225 3.93425 4.89014 5.00747 4.84511 6.85221 5.72058 33 M - - -

2.68618 4.42225 2.77519 2.73123 3.46354 2.40513 3.72494 3.29354 2.67741 2.69355 4.24690 2.90347 2.73739 3.18146 2.89801 2.37887 2.77519 2.98518 4.58477 3.61503

0.00482 5.73338 6.45573 0.61958 0.77255 0.48576 0.95510

33 2.65392 5.71849 3.57348 3.01287 5.05013 2.70660 4.25181 4.51297 1.52246 2.81088 4.77551 3.55118 4.46065 3.35848 1.64188 2.34463 2.62405 4.10465 6.16813 4.78361 34 k - - -

2.68618 4.42225 2.77519 2.73123 3.46354 2.40513 3.72494 3.29354 2.67741 2.69355 4.24690 2.90347 2.73739 3.18146 2.89801 2.37887 2.77519 2.98518 4.58477 3.61503

0.00482 5.73338 6.45573 0.61958 0.77255 0.48576 0.95510

34 2.44771 5.72119 3.54671 2.48927 5.04567 4.05405 2.89359 2.20400 1.16625 4.01436 4.76882 3.53158 4.44647 2.61834 3.44152 3.24865 2.83058 4.10100 6.16337 4.77295 35 k - - -

2.68618 4.42225 2.77519 2.73123 3.46354 2.40513 3.72494 3.29354 2.67741 2.69355 4.24690 2.90347 2.73739 3.18146 2.89801 2.37887 2.77519 2.98518 4.58477 3.61503

0.00482 5.73338 6.45573 0.61958 0.77255 0.48576 0.95510

35 5.16872 6.26109 7.18675 6.65047 4.25555 6.76767 7.07020 1.39797 6.56669 0.41361 3.95860 6.99934 6.52741 6.20139 6.40712 6.29436 5.35764 3.55650 6.68592 5.83910 36 L - - -

2.68618 4.42225 2.77519 2.73123 3.46354 2.40513 3.72494 3.29354 2.67741 2.69355 4.24690 2.90347 2.73739 3.18146 2.89801 2.37887 2.77519 2.98518 4.58477 3.61503

0.00482 5.73338 6.45573 0.61958 0.77255 0.48576 0.95510

36 1.75097 5.45130 4.08048 3.58261 5.03883 2.88539 4.74047 4.45724 3.58208 2.55677 4.89943 4.00997 4.70994 2.40457 4.00946 0.92470 3.71856 4.06676 6.33031 5.03377 37 s - - -

2.68618 4.42225 2.77519 2.73123 3.46354 2.40513 3.72494 3.29354 2.67741 2.69355 4.24690 2.90347 2.73739 3.18146 2.89801 2.37887 2.77519 2.98518 4.58477 3.61503

0.00482 5.73338 6.45573 0.61958 0.77255 0.48576 0.95510

37 1.73990 5.69754 2.70094 2.01760 5.01611 4.03581 4.22696 4.48300 1.62658 2.59181 4.74575 3.51441 4.42943 3.33278 3.46092 3.22830 2.62280 2.83287 6.14678 4.75249 38 k - - -

2.68618 4.42225 2.77519 2.73123 3.46354 2.40513 3.72494 3.29354 2.67741 2.69355 4.24690 2.90347 2.73739 3.18146 2.89801 2.37887 2.77519 2.98518 4.58477 3.61503

0.00482 5.73338 6.45573 0.61958 0.77255 0.48576 0.95510

38 3.36440 5.88648 1.56959 1.67908 5.23214 2.78812 4.30233 4.72065 2.31323 4.18879 4.93135 3.54923 4.50285 2.32075 2.02087 3.31802 3.59820 4.27831 6.31233 4.89638 39 d - - -

2.68618 4.42225 2.77519 2.73123 3.46354 2.40513 3.72494 3.29354 2.67741 2.69355 4.24690 2.90347 2.73739 3.18146 2.89801 2.37887 2.77519 2.98518 4.58477 3.61503

0.00482 5.73338 6.45573 0.61958 0.77255 0.48576 0.95510

39 6.02953 6.77512 6.64960 6.71904 1.00579 6.31120 4.78526 5.53346 6.47265 4.56694 6.00816 5.76191 6.53925 5.97703 6.17266 5.73639 6.19877 5.51130 1.25098 1.22097 40 f - - -

2.68618 4.42225 2.77519 2.73123 3.46354 2.40513 3.72494 3.29354 2.67741 2.69355 4.24690 2.90347 2.73739 3.18146 2.89801 2.37887 2.77519 2.98518 4.58477 3.61503

0.00482 5.73338 6.45573 0.61958 0.77255 0.48576 0.95510

40 1.95473 5.70355 3.59586 3.03230 3.35886 4.08353 2.35250 4.47212 1.58522 3.98881 4.75509 3.56790 4.47338 2.48000 2.01566 3.28135 3.52756 2.89912 6.14847 4.77344 41 k - - -

2.68618 4.42225 2.77519 2.73123 3.46354 2.40513 3.72494 3.29354 2.67741 2.69355 4.24690 2.90347 2.73739 3.18146 2.89801 2.37887 2.77519 2.98518 4.58477 3.61503

0.00482 5.73338 6.45573 0.61958 0.77255 0.48576 0.95510

41 2.35863 5.75329 2.65212 1.74413 5.09655 4.02110 4.20848 4.58123 1.89535 3.02809 4.79446 2.63791 4.41466 3.30543 2.66481 2.67310 3.47811 2.29677 6.18701 4.77717 42 e - - -

2.68618 4.42225 2.77519 2.73123 3.46354 2.40513 3.72494 3.29354 2.67741 2.69355 4.24690 2.90347 2.73739 3.18146 2.89801 2.37887 2.77519 2.98518 4.58477 3.61503

0.00482 5.73338 6.45573 0.61958 0.77255 0.48576 0.95510

42 2.63994 5.06189 4.11021 3.54385 2.90970 4.25960 2.77605 2.62984 2.09143 1.63853 4.16241 3.96746 4.64013 1.96476 3.87019 3.49898 2.66724 3.33927 5.63744 4.39513 43 l - - -

2.68618 4.42225 2.77519 2.73123 3.46354 2.40513 3.72494 3.29354 2.67741 2.69355 4.24690 2.90347 2.73739 3.18146 2.89801 2.37887 2.77519 2.98518 4.58477 3.61503

0.00482 5.73338 6.45573 0.61958 0.77255 0.48576 0.95510

43 3.40740 5.89308 3.58928 2.22905 5.25538 4.14025 4.31820 4.72727 0.93863 4.19115 4.94243 3.60953 4.54385 2.54861 3.43137 2.54579 2.09790 4.29582 6.30459 4.91788 44 k - - -

2.68618 4.42225 2.77519 2.73123 3.46354 2.40513 3.72494 3.29354 2.67741 2.69355 4.24690 2.90347 2.73739 3.18146 2.89801 2.37887 2.77519 2.98518 4.58477 3.61503

0.00482 5.73338 6.45573 0.61958 0.77255 0.48576 0.95510

44 2.64559 5.70871 2.69935 2.37047 5.03191 2.30364 4.22289 2.69696 2.96655 2.43074 4.75532 3.50918 4.42632 1.62490 2.38933 3.22448 3.48296 4.08755 6.15456 4.75727 45 q - - -

2.68618 4.42225 2.77519 2.73123 3.46354 2.40513 3.72494 3.29354 2.67741 2.69355 4.24690 2.90347 2.73739 3.18146 2.89801 2.37887 2.77519 2.98518 4.58477 3.61503

0.00482 5.73338 6.45573 0.61958 0.77255 0.48576 0.95510

45 2.22166 5.75507 2.44878 2.94345 5.09826 4.02305 4.21047 4.58262 2.36114 2.07550 4.79639 1.96446 4.41683 2.54814 2.52528 2.25831 3.48065 4.14474 6.18864 4.77920 46 n - - -

2.68618 4.42225 2.77519 2.73123 3.46354 2.40513 3.72494 3.29354 2.67741 2.69355 4.24690 2.90347 2.73739 3.18146 2.89801 2.37887 2.77519 2.98518 4.58477 3.61503

0.14311 5.73338 2.03941 0.61958 0.77255 0.48576 0.95510

46 2.36587 5.67767 3.41591 2.39741 5.02282 2.32569 4.12779 4.50866 1.94639 2.83692 4.71837 2.55779 2.84547 2.66928 2.34733 2.59303 3.39841 4.06837 6.11019 4.69946 47 k - - -

2.68618 4.42225 2.77519 2.73123 3.46354 2.40513 3.72494 3.29354 2.67741 2.69355 4.24690 2.90347 2.73739 3.18146 2.89801 2.37887 2.77519 2.98518 4.58477 3.61503

0.00553 5.59581 6.31815 0.61958 0.77255 1.04802 0.43175

47 1.96837 5.70815 2.69295 2.13156 5.05327 2.46859 4.14836 4.54113 2.29375 4.01346 4.74965 2.13821 2.83293 2.19030 3.38349 3.15138 3.42383 4.09898 6.13992 4.72587 48 a - - -

2.68618 4.42225 2.77519 2.73123 3.46354 2.40513 3.72494 3.29354 2.67741 2.69355 4.24690 2.90347 2.73739 3.18146 2.89801 2.37887 2.77519 2.98518 4.58477 3.61503

0.00553 5.59581 6.31815 0.61958 0.77255 1.04802 0.43175

48 2.36529 5.65568 3.42486 1.92258 4.99150 2.91600 4.13262 3.19463 2.14216 3.95702 4.69882 2.61149 4.33784 3.23199 2.59455 2.22220 2.54370 2.79438 6.09398 4.68872 49 e - - -

2.68618 4.42225 2.77519 2.73123 3.46354 2.40513 3.72494 3.29354 2.67741 2.69355 4.24690 2.90347 2.73739 3.18146 2.89801 2.37887 2.77519 2.98518 4.58477 3.61503

0.20356 5.59581 1.71221 0.61958 0.77255 1.04802 0.43175

49 2.44120 4.91046 3.83734 1.86911 4.06453 4.03805 4.28012 2.46545 3.24299 1.84922 4.00951 2.55458 4.42098 3.55983 3.63098 2.38381 3.32809 2.40647 5.48184 4.22938 50 l - - -

2.68618 4.42225 2.77519 2.73123 3.46354 2.40513 3.72494 3.29354 2.67741 2.69355 4.24690 2.90347 2.73739 3.18146 2.89801 2.37887 2.77519 2.98518 4.58477 3.61503

0.00454 5.39679 * 0.61958 0.77255 0.00000 *

//
