# Supplementary material for: A widely employed germ cell marker is an ancient disordered protein with reproductive functions in diverse eukaryotes
Source: eLife. 2016 Oct 8;5:e19993. doi: 10.7554/eLife.19993 (PMC5098910; doi:10.7554/eLife.19993)
Supplement: Source code 1. — DOI: http://dx.doi.org/10.7554/eLife.19993.027 [file elife-19993-code1.docx]

#!/usr/bin/perl

#takes protein sequence in fasta format; $ARGV[0] is a file name.
open(IN, "$ARGV[0]") || die "Cannot open protein file\n";
$_ = <IN>; chop; $name = substr($_, 1);
while(<IN>){
     chop; $seq .= $_;
}
close IN;

$ip = get_ip($seq);
print "$name\t$ip\n";

sub get_ip{
#actually calculates isoelectric point
        my($protein) = $_[0];
        $num{D} = $num{E} = $num{C} = $num{Y} = $num{H} = $num{K} = $num{R} = 0;
        $ind{D} = $ind{E} = $ind{C} = $ind{Y} = $ind{H} = $ind{K} = $ind{R} = 1;

        @amino = split '', $protein; $n = @amino;

        for $i (0..$n-1){
                if($ind{$amino[$i]}){
                        $num{$amino[$i]}++;
                }
        }

        $QN1 = $QN2 = $QN3 = $QN4 = $QN5 = $QP1 = $QP2 = $QP3 = $QP4 = $pH0 = 0;
        $NQ = 1;
        $lg = log(10); $pH1 = 14;

     M: $pH = 0.5 * ($pH0 + $pH1);
        $QN1 = -1/(1 + exp($lg * (3.6- $pH)));
        $QN2 = -$num{D}/(1 + exp($lg * (3.9 - $pH)));
        $QN3 = -$num{E}/(1 + exp($lg*(4.1 - $pH)));
        $QN4 = -$num{C}/(1 + exp($lg*(8.5 - $pH)));
        $QN5 = -$num{Y}/(1 + exp($lg*(10.1 - $pH)));
        $QP1 = $num{H}/(1 + exp($lg*($pH - 6.5)));
        $QP2 = 1/(1 + exp($lg*($pH - 8.6)));
        $QP3 = $num{K}/(1 + exp($lg*($pH- 10.8)));
        $QP4 = $num{R}/(1 + exp($lg*($pH - 12.5)));

        $NQ = $QN1 + $QN2 + $QN3 + $QN4 + $QN5 + $QP1 + $QP2 + $QP3 + $QP4;

        if($NQ > 0){
                $pH0 = $pH;
        }
        else{
                $pH1 = $pH;
        }
        if($pH1 - $pH0 < 0.01){
                $pH = 0.5 * ($pH0 + $pH1); return $pH;
        }
        else{ goto M;}

}
